# Long-term efficacy and safety of tonic motor activation for treatment of medication-refractory restless legs syndrome: A 24-Week Open-Label Extension Study

**DOI:** 10.1093/sleep/zsad188

**Published:** 2023-07-13

**Authors:** Asim Roy, Joseph Ojile, Jerrold Kram, Jonathan Olin, Russell Rosenberg, J Douglas Hudson, Richard K Bogan, Jonathan D Charlesworth

**Affiliations:** Ohio Sleep Medicine Institute, Dublin, OH, USA; Clayton Sleep Institute, LLC, St. Louis, MO, USA; California Center for Sleep Disorders, San Leandro, CA, USA; Delta Waves, Inc., Colorado Springs, CO, USA; NeuroTrials Research Inc., Atlanta, GA, USA; FutureSearch Trials of Neurology, Austin, TX, USA; Bogan Sleep Consultants, LLC, Columbia, SC, USA; Noctrix Health, Inc., Department of Clinical Research, Pleasanton, CA, USA

**Keywords:** bioelectronic, neurological disorder, neuromodulation, peripheral nerve stimulation, restless legs syndrome, sleep disorder

## Abstract

**Study Objectives:**

To evaluate long-term efficacy and safety of tonic motor activation (TOMAC) for treatment of medication-refractory moderate-to-severe primary restless legs syndrome (RLS).

**Methods:**

In the parent study (RESTFUL), adults with refractory RLS were randomized to active TOMAC or sham for 4 weeks followed by 4 weeks of open-label active TOMAC. In the extension study, earlier RESTFUL completers comprised the control group (*n* = 59), which was followed for 24 weeks with no TOMAC intervention, and later RESTFUL completers compromised the treatment group (*n* = 44), which received 24 additional weeks of open-label active TOMAC followed by no intervention for 8 weeks. The primary endpoint was Clinician Global Impressions-Improvement (CGI-I) responder rate at week 24 compared to RESTFUL entry.

**Results:**

CGI-I responder rate improved from 63.6% (95% CI, 49.4 to 77.9%) at RESTFUL completion to 72.7% (95% CI, 58.2 to 83.7%) at week 24 for the treatment group versus 13.6% (95% CI, 7.0 to 24.5%) at week 24 for the control group (*p* < 0.0001). Mean change in International RLS Rating Scale (IRLS) score improved from −7.4 (95% CI, −5.6 to −9.2) at RESTFUL completion to -11.3 points (95% CI, −8.8 to −13.9) at week 24 for the treatment group versus −5.4 (95% CI, −3.7 to −7.2) at week 24 for control group (*p* = 0.0001). All efficacy endpoints partially reverted during cessation of treatment. There were no grade 2 or higher device-related adverse events.

**Conclusions:**

TOMAC remained safe and efficacious for >24 total weeks of treatment with partial reversion of benefits upon cessation.

**Clinical Trial:**

Extension Study Evaluating NTX100 Neuromodulation System for Medication-Refractory Primary RLS; clinicaltrials.gov/ct2/show/NCT05196828; Registered at ClinicalTrials.gov with the identifier number NCT05196828.

## Graphical Abstract

**Graphical Abstract:**
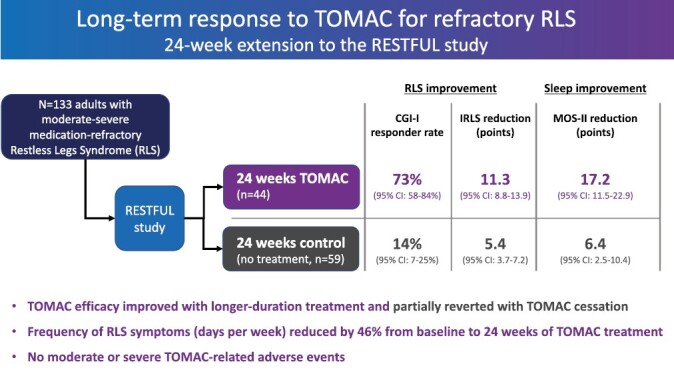

**Statement of Significance**

Tonic motor activation (TOMAC) is a novel wearable therapeutic device that reduces restless legs syndrome (RLS) symptoms. Previous research has demonstrated the short-term efficacy of TOMAC for the treatment of primary moderate-to-severe medication-refractory RLS. This paper presents results from an extension study showing that longer-duration TOMAC treatment over 28–32 weeks provides robust and durable benefits at very low risk to a difficult-to-treat population in need of new therapeutic options. Future research should be directed at understanding the mechanism for why long-term TOMAC use results in reduced RLS symptom frequency and investigating whether TOMAC has similar benefits earlier in the continuum of care, such as for medication-naïve RLS.

## Introduction

Restless legs syndrome (RLS) is a neurological and sleep disorder characterized by uncomfortable leg sensations and an irresistible urge to move the legs [1]. RLS symptoms typically occur in the evening and at night—during rest or inactivity—and result in sleep disturbances [2]. RLS is associated with daytime sleepiness, reduced quality of life, impaired work performance, memory, and cognitive functioning, and increased risk for suicide, self-harm, and medical comorbidities [3–6]. An estimated 2%–3% of adults in the United States and Europe suffer from moderate-to-severe RLS [7].

At least one-third of people with RLS do not have an adequate response to first-line therapeutic agents, dopamine agonists (DAs), and alpha-2-delta ligands, and an additional 5%–20% experience intolerable side effects or augmentation, defined as paradoxical increases in RLS symptoms relative to natural progression of the disorder, with long-term use of DAs [8]. Given that no new drug or device therapies have been approved for treatment of RLS since DAs and alpha-2-delta ligands were introduced over a decade ago, there is a significant unmet need for effective and safe treatments for patients who are refractory to RLS medication and continue to suffer from moderate-to-severe RLS symptoms.

Recognizing the unmet clinical need, in 2020, the US Food and Drug Administration granted a Breakthrough Device designation for an investigational therapeutic device, tonic motor activation (TOMAC) [9] for the treatment of primary moderate-to-severe medication-refractory RLS. The TOMAC device is positioned over the head of the fibula bone and stimulates afferent peroneal nerve fibers to evoke sustained increases in tibialis anterior muscle tone without inducing leg movements that could be disruptive to sleep [10]. Through this mechanism, TOMAC engages the neuromuscular pathways associated with voluntary leg movements to provide sleep-compatible reduction of RLS symptoms. The advantages of TOMAC over voluntary leg movements include sleep compatibility, more efficacious relief of severe symptoms, and longer duration of therapeutic relief [10]. TOMAC can be administered by the patient whenever symptoms present either during the sleep period, at bedtime, or during waking hours.

The efficacy and safety of TOMAC have been demonstrated in two randomized sham-controlled clinical trials with a maximum of 8 weeks of TOMAC treatment. The first of these trials enrolled a mixture of medication-refractory and medication-naïve adults with primary moderate-to-severe RLS who completed 2 weeks of TOMAC treatment and 2 weeks of sham control in a crossover design [11]. The second trial (RESTFUL study)—which is the parent study of this extension study—enrolled medication-refractory adults with primary moderate-to-severe RLS who were randomly assigned to 4 weeks of active or sham TOMAC treatment (stage 1) followed by 4 weeks of open-label active TOMAC (stage 2) [12]. Both studies showed that TOMAC substantially improved RLS symptoms relative to sham control and was well tolerated and safe.

The primary objective of this extension study was to assess the longer-term efficacy and safety of TOMAC over 24 weeks for the treatment of medication-refractory moderate-to-severe primary RLS. A secondary objective of this extension study was to characterize changes to RLS symptoms following cessation of TOMAC treatment.

## Methods

### Study design

This study was a multicenter, prospective, open-label extension to the RESTFUL study, the results of which are published separately [12]. The RESTFUL study (NCT04874155) was a prospective, randomized, double-blind, sham-controlled pivotal trial conducted at 7 sleep clinics in the United States. In the RESTFUL study, participants were randomized 1:1 to active or sham TOMAC for a double-blind, 4-week stage 1 and all received active TOMAC during open-label, 4-week stage 2. During stage 1 (weeks 1–4), participants were randomized 1:1 to active or sham TOMAC treatment. There was no drug washout; participants were required to maintain a stable dose and schedule of RLS medication for the 30 days prior to RESTFUL study entry and throughout the duration of the study. Details of the randomization and masking process are described in the RESTFUL publication [12].

The initiation of this extension study was contingent on obtaining a sufficient supply of TOMAC system components. As a result of global shortages during the coronavirus disease 2019 pandemic, these supplies were not available until December 2021, whereas the RESTFUL study began enrollment in May 2021. Each of the seven clinical centers from the RESTFUL study participated in the extension study with agreement dates ranging from December 2021 to January 2022.

All RESTFUL study completers were eligible unless they failed to comply with RESTFUL study requirements; this resulted in ineligibility of 1 potential participant for treatment group (participant changed RLS medication dosage) and 0 participants for control group. Enrollment in the treatment group required direct rollover to continued TOMAC treatment and thus recruited all participants who completed the RESTFUL study after initiation of the extension study at a given site; all participants in the treatment group consented and enrolled on the day of RESTFUL study completion. All participants who completed the RESTFUL study prior to the initiation of the extension study at a given site or who did not consent to the treatment group were eligible for the control group; all control group participants enrolled at 0, 8, 16, or 24 weeks after RESTFUL study completion. Participants who completed the RESTFUL study >24 weeks before the extension study began were excluded from the control group.

The treatment group received 24 weeks of active TOMAC treatment (Weeks 1-24) followed by 8 weeks without TOMAC treatment (weeks 24–32) to evaluate response to cessation of TOMAC treatment. Participants taking concurrent RLS medications at study entry were required to maintain a stable dosage and schedule through week 32. The control group received 24 weeks of standard of care with no TOMAC intervention; there were no limitations regarding changes to RLS medication for these participants.

The study was conducted in accordance with the International Conference on Harmonization guidelines on good clinical practice and the Declaration of Helsinki, the protocol and informed consent were approved by a central institutional review board (Advarra, Columbia, MD, USA), and all participants provided informed consent. The first participant was enrolled on December 20, 2021, and the trial was registered with ClinicalTrials.gov (NCT05196828) on December 31, 2021, within the 21-day window permitted by 42 CRF Part 11. The study protocol is available as Supplementary Material.

### Participants

RESTFUL study completers were eligible unless they failed to comply with RESTFUL study instructions or developed a new contraindication to TOMAC. The RESTFUL study enrolled individuals aged 22–79 years with medication-refractory moderate-to-severe primary RLS. Consistent with the most recent expert consensus definition [13] and the TOMAC indication from the US Food and Drug Administration Breakthrough Device designation, medication-refractory RLS was defined as having failed one or more medications commonly prescribed to treat RLS (ropinirole, pramipexole, gabapentin, pregabalin, gabapentin enacarbil, and/or rotigotine) for at least one of the following reasons: intolerable adverse effects, symptoms of augmentation, up-titration needed due to reduced efficacy, and insufficient response at maximum approved, recommended, or tolerated dosage. RESTFUL study key inclusion criteria included International RLS Study Group Rating Scale (IRLS) total score ≥15, symptoms ≥2 nights per week, symptoms most significant in the lower legs and/or feet, and symptoms most significant at bedtime, after bedtime, and/or in the 2 hours before bedtime. Parent study key exclusion criteria were unstable doses (e.g. as needed doses) of RLS medications, sleep medications, or antidepressants, inadequately treated primary sleep disorders other than RLS (e.g. nonadherence to CPAP for moderate–severe obstructive sleep apnea), severe peripheral neuropathy affecting the lower legs, skin conditions affecting the application site, severe RLS symptoms between 10:00 am and 06:00 pm, known allergy to device materials, active medical device implants, epilepsy, dialysis, and iron-deficient anemia, and prior experience with the study device or with any neurostimulation device to treat RLS.

### Procedures

**Figure 1.**
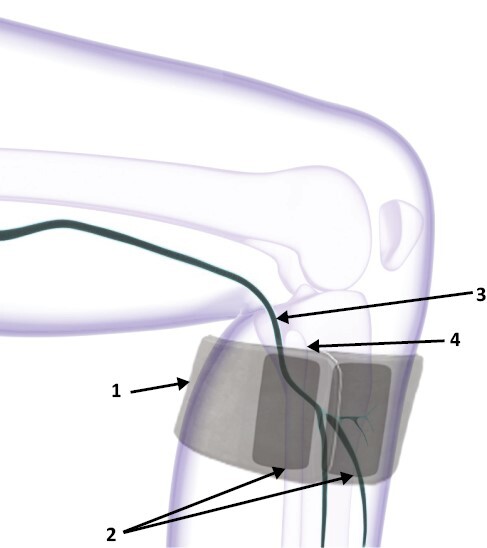
Anatomical Positioning of TOMAC Therapy Unit. Depiction of a (1) TOMAC therapy unit attached to the right leg, with (2) charge-dispersing interfaces positioned over the (3) peroneal nerve, distal to the (4) head of the fibula. The TOMAC system consists of two therapy units, one attached to each leg.

The TOMAC system was comprised of two therapy units worn bilaterally on the lower legs (Figure 1). TOMAC modulates afferent fibers of the peroneal nerve to evoke tonic motor activation of the tibialis anterior muscle, thereby engaging the neuromuscular circuits associated with voluntary leg movements without triggering leg movements [10]. Participants were trained to position therapy units so that the charge-dispersing interfaces covered the peroneal nerve at the head of the fibula bone. Participants were instructed to self-administer a therapy session whenever they experienced distressing RLS symptoms while prioritizing usage at bedtime, after bedtime, or in the 2 hours before bedtime, and to use a maximum of four sessions (120 minutes) per day. When activated by the user, the therapy units transmit a continuous 4000 Hz waveform for 30 minutes, after which stimulation automatically shuts off. A previously described [10] titration procedure was used to individualize the stimulation intensity default value within a range of 15 to 40 mA; from this default value, participants could increase or decrease intensity by 0–4 mA.

### Outcomes

Outcome measures were listed in prespecified order to first evaluate treatment group Week 24 relative to baseline (primary endpoint and secondary endpoints 1–5) and then evaluate treatment group week 24 relative to control group week 24 (secondary endpoints 6-11). The primary endpoint was the Clinical Global Impressions-Improvement (CGI-I) responder rate at week 24 for treatment group, with responders defined as “much improved” or “very much improved” relative to baseline (i.e. entry to the RESTFUL study). Key secondary endpoints 1–5 were assessed at week 24 for the treatment group relative to baseline and ordered as follows: Patient-Global Impressions-Improvement (PGI-I) responder rate, change in total IRLS score, change in Medical Outcomes Study Sleep Problem Indices II (MOS-II) and I (MOS-I), and change in IRLS Question #7 score (frequency of RLS symptoms). Key secondary endpoints comparing the treatment and control groups at week 24 were assessed in the same order (CGI-I, PGI-I, IRLS, MOS-II, MOS-I, and IRLS Question #7). The primary endpoint and key secondary endpoints comparing week 24 of the treatment group relative to baseline were prespecified in the study protocol; additional key secondary endpoints were prespecified in the statistical analysis plan prior to database lock.

### Statistical analysis

The sample size for this study was not prespecified; all participants who completed the RESTFUL study could enroll if they met the eligibility criteria. Efficacy endpoints were tested hierarchically until failure in the prespecified order listed above. Efficacy outcome measures were centrally assessed for the intent-to-treat population, which comprised all eligible participants who passed screening and were enrolled. Last observation carried forward imputation was used for all efficacy endpoints. For one-sample proportions (CGI-I and PGI-I responder rates for the treatment group), there was no appropriate reference value and thus 95% confidence intervals were presented instead of hypothesis testing. For all other endpoints, hierarchical hypothesis testing was employed to account for multiplicity. Mean scores were compared using *t*-tests and two-sample proportions were compared using normal approximation tests; a one-sided alpha level of 0.025 defined statistical significance for all comparisons. For comparisons of outcome measures between week 24 and baseline for the treatment group, effect sizes were calculated based on Cohen’s d for means and Cohen’s h for proportions; these metrics evaluate the magnitude of difference irrespective of sample size. Statistical analyses were conducted using R version 4.2.2. The stable medication population included all treatment group participants who did not add, discontinue, or change dosage of prescription RLS medication. For calculating the number of days per week with RLS symptoms, each IRLS question 7 response was converted to a frequency; for example, responses of “Severe (This means 4 to 5 days a week)” were scored as 4.5 days per week.

The prespecified analysis described above evaluated the cross-sectional effect of TOMAC at week 24. It was also of interest to conduct exploratory analysis measuring the effect of TOMAC over the entire duration of treatment in the extension study based on repeated measures at weeks 8, 16, and 24 compared to week 0. Generalized linear models in SPSS version 29.0 were employed for repeated measures comparisons between treatment group and control group (to calculate treatment effect and treatment-time interaction) and between treatment group and baseline (to calculate time effect). A one-sided alpha level of 0.025 defined statistical significance for all comparisons.

Differences in participant characteristics between treatment and control groups were evaluated using *t*-tests for continuous variables (e.g. age) and Fisher’s exact test for categorical variables (e.g. race/ethnicity).

### Safety analysis

Safety outcome measures were centrally assessed. The count and proportion of participants reporting adverse events (AEs) with new onset or worsening severity (relative to baseline) were compared between treatment groups and stages. AEs were coded and summarized at the participant level by Medical Dictionary for Regulatory Activities system organ class and preferred term and by seriousness, severity, and relationship to the device.

The safety analysis population included all participants. Rates of AEs were compared using one-sided, two-sample *t*-tests with no accounting for multiplicity.

Each therapy unit included an electronic data logger that tracked the intensity level of each completed TOMAC session. For each week, stimulation intensities were averaged within participants (across all sessions for that participant for that week) prior to calculating statistics across participants.

### Data availability

The deidentified data that support the findings of this study are available from the corresponding author upon reasonable request.

## Results

### Participant characteristics and disposition

**Figure 2.**
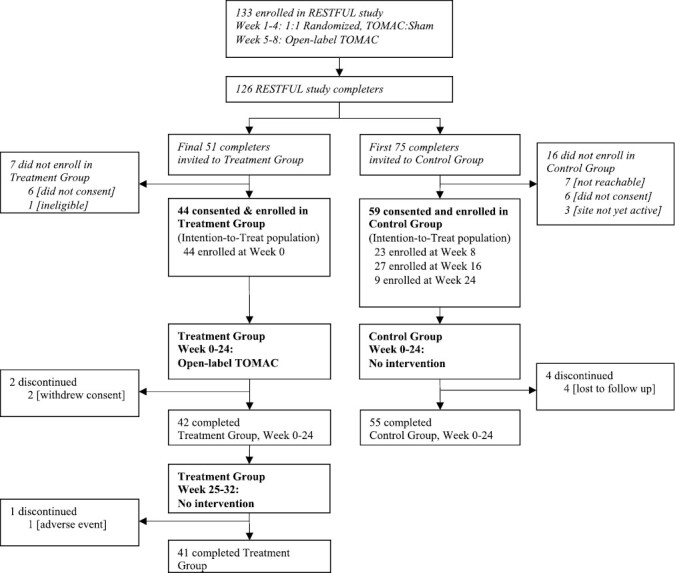
Participant disposition.

Subject disposition is shown in Figure 2. The RESTFUL study consisted of a 4-week randomized, sham-controlled trial followed by 4 weeks of open-label TOMAC. The final 51 RESTFUL study completers were recruited for direct rollover into the treatment group of the extension study; 45 (88%) consented and 44 were enrolled. The first 75 RESTFUL study completers were recruited for the control group, consisting of symptom evaluation with no intervention; 59 consented and were enrolled.

Participant characteristics are shown in Table 1. Although assignment to the treatment or control group was based on timing of parent study completion instead of randomization, participant characteristics were largely similar between the two group. The majority of participants in both groups were female and white, non-Hispanic, with a mean age of about 57 years and a mean duration of RLS symptoms of 22 years. Participants in both groups had average RLS symptoms in the severe range at RESTFUL study entry and in the moderate range upon completion of RESTFUL.

**Table 1.** Participant Characteristics

	Treatment group (*n* = 44)	Control group (*n* = 59)	*P*-value
Age at entry to parent study, mean (SD), y	57.7 (11.65)	57.5 (11.85)	0.93
Sex, *n* (%)			0.69
Male	18 (40.9)	27 (45.8)	
Female	26 (59.1)	32 (54.2)	
Race/Ethnicity, *n* (%)			0.46
White/Hispanic or Latino	4 (9.1)	3 (5.1)	
White/Not Hispanic or Latino	40 (90.9)	52 (88.1)	
Asian	0 (0.0)	2 (3.4)	
Black or African American	0 (0.0)	2 (3.4)	
IRLS total score at parent study entry, mean (SD)	24.5 (5.27)	25.8 (5.15)	0.32
IRLS total score at parent study completion, mean (SD)	17.1 (6.75)	17.0 (7.65)	0.77
Duration of RLS symptoms, mean (SD), y	22.1 (13.98)	22.5 (16.00)	0.90
Refractory RLS medications, *n* (%)			0.43
Dopamine agonists	39 (88.6)	49 (83.1)	
Alpha-2-delta ligands	19 (43.2)	36 (61.0)	
Both	14 (31.8)	26 (44.1)	
Categories of current RLS medication, *n* (%)			0.83
Dopamine agonists	31 (70.5)	39 (66.1)	
Alpha-2-delta ligands	12 (27.3)	17 (28.8)	
Opioids	4 (9.1)	2 (3.4)	
Benzodiazepines	1 (2.3)	0 (0.0)	
Other	4 (9.1)	5 (8.5)	
No prescribed RLS medications	7 (15.9)	9 (15.3)	

Abbreviations: IRLS, International Restless Legs Syndrome Study Group Rating Scale; SD, standard deviation.

### Efficacy results for treatment group compared to baseline

Table 2 summarizes the primary and key secondary endpoints for the treatment group, each of which were measured at wek 24 relative to baseline (RESTFUL study entry). At week 24, 72.7% of participants were CGI-I responders (primary endpoint; 95% CI, 58.2 to 83.7) and 75.0% were PGI-I responders (95% CI, 60.6 to 85.4). The mean change in IRLS total score was −11.3 points (*p* < 0.0001, 95% CI: −8.8 to −13.9), from 24.5 points at baseline to 13.2 points at week 24. On the MOS sleep scale, the MOS-II score improved from 44.8 to 27.7 (change of −17.2, *p* < 0.0001, 95% CI: −11.5 to −22.9) and MOS-I improved from 41.4 to 25.5 (change of −15.8, *p* < 0.0001, 95% CI: −10.0 to −21.7). RLS symptom frequency reduced substantially; IRLS question 7 score improved from 3.7 to 2.2 (change of −1.50, *p* < 0.0001, 95% CI: −1.09 to −1.91), corresponding to a 46 percent reduction in the frequency of RLS symptoms from 5.9 days/week to 3.2 days/week. At week 24, 29.5% of treatment group participants (13 of the 44) reported RLS symptoms on one or fewer days per week compared to 0% at RESTFUL study entry.

**Table 2.** Primary and Key Secondary Endpoints

	Statistic	Treatment group vs. baseline	Control group vs. baseline	*P*-value, treatment vs. baseline	*P*-value, treatment vs. Control	Effect size, treatment vs. Control
CGI-I Responder						
	*n*	44	59			
	Responder %	72.7	13.6	NA	<0.0001	1.29
	Responder *n*	32	8			
	95% CI	58.2 to 83.7	7.0 to 24.5			
PGI-I Responder						
	*n*	44	59			
	Responder %	75.0	20.3	NA	<0.0001	1.16
	Responder *n*	33	12			
	95% CI	60.6 to 85.4	12.0 to 32.3			
Change in IRLS total score						
	*n*	44	59			
	Mean	−11.3	−5.4	<0.0001	0.0001	0.70
	Median	−9.0	−4.0			
	SD	8.43	6.70			
	Min, Max	−29, −9	−23, −10			
	95% CI	−13.9 to −8.8	−3.7 to −7.2			
Change in MOS-II score						
	*n*	44	59			
	Mean	−17.2	−6.4	<0.0001	0.0013	0.57
	Median	−13.3	−6.1			
	SD	18.81	15.18			
	Min, Max	−68, −12	−50, −27			
	95% CI	−22.9 to −11.5	−10.4 to −2.5			
Change in MOS-I score						
	*n*	44	59			
	Mean	−15.8	−5.7	<0.0001	0.0026	0.53
	Median	−13.3	−6.7			
	SD	19.21	15.48			
	Min, Max	−60, −20	−43, −27			
	95% CI	−21.7 to −10.0	−9.7 to −1.7			
Change in IRLS Question 7 score						
	*n*	44	59			
	Mean	−1.50	−0.41	<0.0001	<0.0001	0.82
	Median	−2.0	0.0			
	SD	1.24	0.91			
	Min, Max	−4, −2	−4, −1			
	95% CI	−1.91 to −1.09	−0.64 to −0.17			

All treatment and control group statistics correspond to week 24 compared to the baseline of RESTFUL study entry.

Abbreviations: CGI-I, Clinical Global Impressions-Improvement; IRLS, International Restless Legs Syndrome Study Group Rating Scale; MOS-I, Medical Outcomes Study Sleep Problem Index I; MOS-II, Medical Outcomes Study Sleep Problem Index II; NA, not applicable; PGI-I, Patient-Global Impressions-Improvement; SD, standard deviation.

### Efficacy results for treatment group compared to control group

Efficacy outcomes were also compared between the treatment and control groups at week 24. The treatment group showed significantly greater improvement on each outcome at week 24 compared with the control group, with moderate-to-large effect sizes ranging from 0.53 for the MOS-I to 1.29 for the CGI-I responder rate (Table 2). At week 24, CGI-I responder rate was 72.7% (95% CI, 58.2 to 83.7) for treatment group compared to 13.6% (95% CI, 7.0 to 24.5) for control group (*p* < 0.0001) and mean change in IRLS score from baseline was −11.3 points (95% CI, −8.8 to −13.9) for treatment group compared to −5.4 points (95% CI, −3.7 to −7.2) for control group (*p* = 0.0001, Table 2).

For the control group, the remaining improvements at week 24 relative to baseline (Table 2) were not attributable to increases in medication dose in this group; the 15 participants who changed medication (CGI-I responder rate: 13.3%, PGI-I responder rate: 13.3%, mean IRLS score change: −2.1) showed less improvement on the PGI-I and IRLS compared to the 44 participants who remained on stable medication (CGI-I responder rate: 13.6%, PGI-I responder rate: 22.7%, mean IRLS score change: −6.5). Among the 15 participants who changed medication, three were taking DAs at study entry, seven were taking alpha-2-delta ligands, one was taking opioids, and two were taking no RLS medication.

### Repeated measures analysis of TOMAC efficacy over time

**Figure 3.**
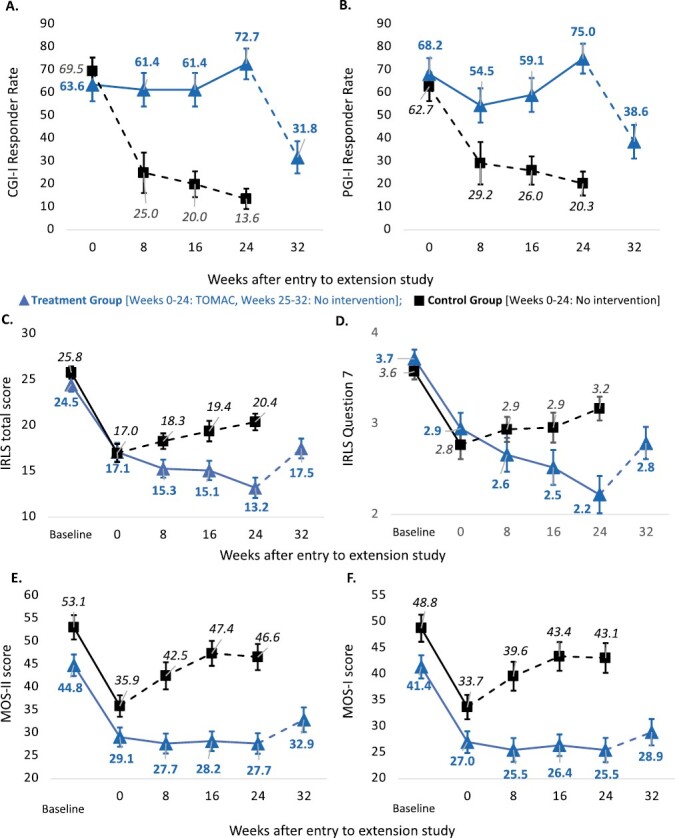
Efficacy results for the intervention and control groups. For each efficacy outcome measure, results are shown at weeks 0, 8, 16, 24, and 32 after entry into the extension study. Results are presented as responder rates relative to baseline for (A) CGI-I and (B) PGI-I and presented as mean scores for (C) IRLS total, (D) IRLS Question 7, (E) MOS-II, and (F) MOS-I. Treatment group data are shown in blue with triangle markers and **bold** data labels. Control group data are shown in black with square markers and data labels in *italics*. For both treatment and control groups, solid lines represent periods with TOMAC, and dashed lines represent periods with no intervention. Error bars represent +/− standard error.

The results above showed that TOMAC was efficacious after 24 weeks of treatment. Next, we evaluated if TOMAC was consistently efficacious over the full 24 weeks of treatment based on analysis of repeated measures at weeks 8, 16, and 24 (Figure 3). Repeated measures analysis demonstrated that the treatment group showed significantly greater improvement on each outcome compared to the control group (Table 3). This analysis also revealed a significant treatment vs. time interaction for CGI-I, PGI-I, IRLS, and IRLS Question 7 (Table 3), indicating that the difference between treatment and control groups increased over time.

**Table 3.** Results of Repeated Measures Analysis

	Treatment group vs. control group, *P*-values		Treatment group vs. baseline, *P*-values
	Treatment effect	Time-treatment interaction	Time effect
CGI-I responder rate	<0.001	<0.001	0.642
PGI-I responder rate	<0.001	0.006	0.193
Change in IRLS total score	<0.001	0.004	0.059
Change in MOS-II score	<0.001	0.045	0.961
Change in MOS-I score	<0.001	0.106	0.51
Change in IRLS question 7 score	0.004	0.009	0.053

Results of generalized linear model comparisons across the repeated measures at weeks 8, 16, and 24 compared to Week 0.

Abbreviations: CGI-I, Clinical Global Impressions-Improvement; IRLS, International Restless Legs Syndrome Study Group Rating Scale; MOS-I, Medical Outcomes Study Sleep Problem Index I; MOS-II, Medical Outcomes Study Sleep Problem Index II.

Within the treatment group, there was also a trend towards progressively increased efficacy with longer-duration treatment from week 0 to 24 during the extension study (Figure 3, blue solid lines). Repeated measures analysis showed that these trends approached statistical significance for IRLS Question 7 score (*p* = 0.053) and IRLS total score (*p* = 0.059) (Table 3). The reduction in IRLS Question 7 score corresponded to a 27 percent reduction in the frequency of RLS symptoms, from 4.4 days per week at RESTFUL study completion (week 0) to 3.2 days per week after 24 additional weeks of treatment (week 24).

To further evaluate the trends towards increased response with longer-duration TOMAC, we measured the effect size from week 0 to week 24 in the treatment group based on Cohen’s d and Cohen’s *h*, metrics which measure standardized differences irrespective of sample size. The largest improvements were observed for IRLS Question 7 score (Cohen’s *d*, 0.74) and total IRLS score (Cohen’s *d*, 0.65); smaller improvements were observed for the CGI-I (Cohen’s *h*, 0.20), PGI-I (Cohen’s *h*, 0.15), MOS-II (Cohen’s *d*, 0.09), and MOS-I (Cohen’s *d*, 0.09). For the CGI-I, PGI-I, IRLS total score, and IRLS Question 7 score, positive trends continued from week 16 to week 24 (Figure 3), raising the possibility that longer use of TOMAC beyond 6 months could result in even greater benefits.

### Response to cessation of TOMAC

Cessation of TOMAC resulted in partial reversion of improvements to RLS symptoms. During the first 8 weeks without TOMAC treatment for the control group (week 0-8; Figure 3, black dashed lines), CGI-I responder rate decreased from 69.5% to 25.0% (−5.6% per week) and IRLS score increased from 17.0 to 18.3 points (+0.2 points per week). During the first 8 weeks without TOMAC treatment for the treatment group (week 24-32; Figure 3, blue dashed lines), CGI-I responder rate decreased from 72.7% to 31.8% (−5.1% per week) and IRLS score increased from 13.2 to 17.5 (+0.5 points per week). Notably, although all outcome measures partially reverted towards baseline, no outcome measures fully reverted to baseline or rebounded past baseline, even after 24 weeks without TOMAC treatment in the control group (Figure 3).

### Safety

AEs were assessed for treatment group during weeks 1–24, the period of the study which involved device usage (Table 4). All device-related AEs were mild (grade 1) and resolved rapidly without medical intervention. There were no serious or severe device-related AEs. No participants discontinued study participation due to an AE. The only category of AEs that occurred in greater than 5% of participants was discomfort (three participants, 6.8%). The frequency of device-related AEs was reduced from 31.8% during the 8-week RESTFUL study to 9.1% during the 24-week extension study and the frequency of discomfort reduced from 22.7% in the RESTFUL study to 6.8% in the extension study.

**Table 4.** Adverse Events in the Treatment Group

	RESTFUL study (*n* = 44)	Extension study (*n* = 44)
*AE*		
Any	21 (47.7%)	14 (31.8%)
Device-related	14 (31.8%)	4 (9.1%)
*Serious AE*		
Any	1 (2.3%)	0
Device-related	0	0
*Discontinuation due to AE*		
Any	0	0
Device-related	0	0
*MedDRA preferred term* ^a^		
Discomfort	10 (22.7%)	3 (6.8%)
*AE severity*		
Grade 1	19 (43.2%)	10 (22.7%)
Grade 2	3 (6.8%)	5 (11.4%)
Grade 3	1 (2.3%)	0
Grade 4 or 5	0	0

^a^MedDRA preferred terms occurring in 5% of more of participants are shown.

Abbreviations: AE, adverse event; MedDRA, Medical Dictionary for Regulatory Activities; TOMAC, tonic motor activation.

### Stimulation Intensity

**Figure 4.**
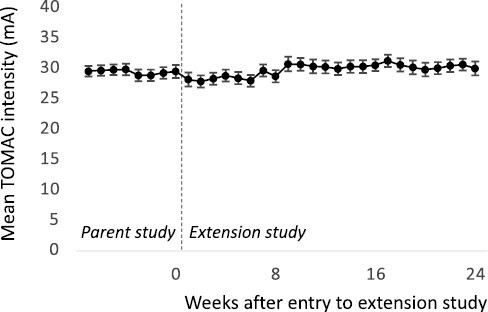
Patient-administered TOMAC Intensity. Each point represents mean patient-administered TOMAC intensity for all treatment group participants (*n* = 44) for a given week in the RESTFUL (parent) study and extension study. Vertical dashed line indicates entry to the extension study. Error bars are +/− SEM.

Across participants in the treatment group, there was no trend towards changes in TOMAC intensity with continued use (Figure 4). Mean (standard error of mean) patient-administered stimulation intensity was 29.6 mA (0.9 mA) at week 1 of the RESTFUL study, 29.5 (1.1 mA) at week 8 of the RESTFUL study, and 30.0 (1.1 mA) at week 24 of the extension study.

## Discussion

TOMAC remained efficacious over an additional 24 weeks of treatment across all outcomes, with a trend toward further improvement with continued TOMAC usage. By the completion of treatment at 24 weeks, nearly three-quarters of participants in the treatment group were CGI-I responders and the mean reduction in IRLS total score was 11.3 points, which was more than double the reduction seen in control group and almost fourfold higher than the minimal clinically significant change of 3 points [14]. The safety of TOMAC with long-term use was excellent; there were no significant device-related AEs and the frequency of each type of device-related AE was reduced relative to the RESTFUL study. There was no evidence of tolerance or reduced benefit over time; participants did not need to increase TOMAC stimulation intensity and TOMAC cessation did not result in worsening of RLS symptoms relative to baseline. In summary, these data demonstrate that the response to TOMAC is robust and durable across long-term treatment and establishes the long-term safety and tolerability of TOMAC.

Study findings suggest that there are persistent benefits of TOMAC that gradually build over many weeks of treatment and do not fully revert even after 24 weeks without TOMAC. The treatment group showed a trend towards an increased response for each outcome measure from extension study entry to week 24. Cessation of TOMAC resulted in gradual and partial reversion of improvement without evidence of rebound or withdrawal. In contrast, DAs, the most common RLS treatment, have high initial efficacy but often result in symptoms of tolerance and dependency (augmentation) [8], such that discontinuation leads to a rebound in RLS symptoms above baseline. These findings suggest a potential advantage of TOMAC over DAs and further point to potential long-lasting benefits of TOMAC.

Long-term TOMAC treatment led to a substantial reduction in the frequency of RLS symptoms. Average frequency of symptoms was reduced from 5.9 to 3.2 days per week, meaning that the number of symptom-free days per week increased from 1.1 to 3.8, a 3.5-fold increase. Importantly, the reduction in the frequency of RLS symptoms conferred by TOMAC continued to improve over each consecutive 8-week period of TOMAC treatment (Figure 3D), raising the question that longer-term use beyond 24 weeks could result in an even greater reduction in symptom frequency.

There are two compelling hypotheses that could explain the reduction in RLS symptoms frequency with long-term TOMAC treatment. First, long-term TOMAC treatment applied when RLS symptoms are present could result in neural plasticity at the spinal or brain level that inhibits or reverses RLS pathophysiology. This phenomenon may be consistent across multiple neurostimulation therapies; implanted neurostimulation therapies for epilepsy result in a similar trend toward increased responder rate with longer-term treatment [15, 16]. Second, improvements in sleep quality that result from TOMAC treatment could result in health benefits that inhibit or reverse RLS pathophysiology. There is a compelling body of research showing that sleep deprivation results in pathophysiological neuronal changes that increase the risk for neurological dysfunction [17, 18]. Consistent with this latter possibility, TOMAC-related improvements to sleep tended to occur and plateau at the end of the RESTFUL study (Figure 3E-F) and before the reduction in RLS symptoms frequency, whereas reductions to RLS symptoms frequency continued throughout the extension study (Figure 3D). These hypotheses are not mutually exclusive and further testing will be needed to determine their relative influence on the benefits of TOMAC.

The results presented here establish the long-term benefits of TOMAC for reducing subjective RLS symptoms and subjective sleep quality. Whereas subjective rating scales are the gold standard for evaluating RLS [19], RLS is also typically associated with periodic leg movements during sleep (PLMS) and wake (PLMW), which can be objectively measured and are reduced by dopaminergic and alpha-2-delta ligand medications [20]. Future research would be needed to determine if TOMAC also results in reductions to PLMS and/or PLMW.

Whereas the treatment group represents a typical open-label extension study design, there are limitations to consider when comparing the results between treatment and control groups. The control group was not a “no treatment” group but represented the current standard of care for refractory RLS; participants in the control group had no restrictions on their RLS medication and were permitted to increase dosage or add medications. This allowance was intended to minimize barriers to enrollment in the control group, which was necessary because the recruitment pool was limited to RESTFUL study completers. Notably, control group participants who changed medication did not show improved RLS symptoms relative to those who continued stable medication. This reflects the “refractory” nature of the patient population in this study and reinforces the substantial unmet need for these patients. Additionally, participants were assigned to groups based on timing of RESTFUL study completion as opposed to randomization. This did not lead to any significant differences in participant characteristics between the treatment and control groups (Table 1). However, it did cause a gap period between RESTFUL study completion and extension study enrollment where control group participants were not being observed by research staff; this gap could have potentially influenced the psychology of the control group compared to the treatment group and thus affected outcomes [21]. Importantly, these limitations do not affect the interpretation of the primary endpoint nor the key secondary endpoints that were specific to the treatment group.

In summary, these results indicate that TOMAC is a promising, noninvasive long-term treatment for medication-refractory moderate-to-severe primary RLS. TOMAC has the potential to provide strong and durable efficacy at very low risk to this difficult-to-treat patient population and serve as a favorable alternative to off-label opioids.

## Supplementary Material

zsad188_suppl_Supplementary_Material
