# Supplementary material for: Long-term efficacy and safety of tonic motor activation for treatment of medication-refractory restless legs syndrome: A 24-Week Open-Label Extension Study
Source: Sleep. 2023 Jul 13;46(10):zsad188. doi: 10.1093/sleep/zsad188 (PMC10566237; doi:10.1093/sleep/zsad188)
Supplement: zsad188_suppl_Supplementary_Material [file zsad188_suppl_supplementary_material.pdf]

Supplementary Material

Manuscript Title: Long-term Efficacy and Safety of Tonic Motor Activation (TOMAC) for Treatment of Medication-Refractory Restless Legs Syndrome: A 24-Week Open-label Extension Study

Authors:
Asim Roy[1]
Joseph Ojile[2]
Jerrold Kram[3]
Jonathan Olin[4]
Russell Rosenberg[5]
J. Douglas Hudson[6]
Richard K. Bogan[7]
Jonathan D. Charlesworth[8]

[1]Ohio Sleep Medicine Institute, Dublin, OH, USA
[2]Clayton Sleep Institute, LLC, St. Louis, MO, USA
[3]California Center for Sleep Disorders, San Leandro, CA, USA
[4]Delta Waves, Inc., Colorado Springs, CO, USA
[5]NeuroTrials Research Inc., Atlanta, GA, USA.
[6]FutureSearch Trials of Neurology, Austin, TX, USA
[7]Bogan Sleep Consultants, LLC, Columbia, SC, USA
[8]Noctrix Health, Inc., Pleasanton, CA, USA

Corresponding Author:
Dr. Jonathan D. Charlesworth
Department of Clinical Research
Noctrix Health, Inc.
6700 Koll Center Pkwy, Ste 310
Pleasanton, CA 94566
Phone: (804) 683-4279
jcharlesworth@noctrixhealth.com

CLINICAL INVESTIGATIONAL PLAN

Open-label Extension Study to Evaluate Longer-Duration Response to the NTX100 Neuromodulation System for Patients with Medication-Refractory Primary Restless Legs Syndrome (RLS)

PROTOCOL NUMBER: CT-05

VERSION: 1.0

SPONSOR:
Noctrix Health, Inc.
5424 Sunol Blvd
Ste 10-451
Pleasanton, CA 94566
804-683-4279

SPONSOR REPRESENTATIVE(S):
Jonathan Charlesworth, PhD
VP of Clinical Research

Maggie LeDang
Clinical Operations Manager

Van Lieou
Sr. Clinical Project Manager

The information contained in this document is confidential and proprietary property of Noctrix Health, Inc.

Investigator Study Acknowledgement

Read and initial below.

____ I understand this protocol contains information that is confidential and proprietary to Noctrix Health, Inc.

____ Any additional information added to this protocol is also confidential and proprietary to Noctrix Health, Inc. and must be treated in the same manner as the contents of this protocol.

____ I have read the entire protocol.

____ I understand what the protocol asks me to do as an Investigator.

____ I will conduct this study following this protocol and will make a reasonable effort to complete the study in the time noted.

____ I will provide this protocol to study staff under my direct supervision. My study staff will keep the protocol and associated documents confidential.

____ I will discuss this information with the study staff to ensure they are fully informed about the study and the test articles.

____ I will not start enrolling in this study until it is approved by a governing Institutional Review Board.

____ I understand the study may be terminated or enrollment suspended at any time by Noctrix Health, Inc., with or without cause, or by me if it becomes necessary to protect the interests of the study subjects.

____
Name of Investigator

____________________________________ ______________________
Investigator Signature Date

CONFIDENTIAL
All information contained in this document is confidential and is the sole property of Noctrix Health, Inc.
Any reproduction in part or whole without the written permission of Noctrix Health, Inc. is prohibited.

Table of Contents

Investigator Study Acknowledgement 2
Study Design Overview Synopsis 5
Abbreviations 7
1. Background 8
2. Regulatory Status 12
3. Objective 12
4. Study Design 12
5. Blinding 14
6. Duration 14
7. Study population overview 14
7.2. Complete list of inclusion criteria (IC) 15
7.3. Complete list of exclusion criteria (EC) 16
7.4. Definition of RLS Refractory to Medication (IC #2) 18
8. Investigational Device 19
9. Risks 22
10. Adverse Events 22
11. Device Tracking 26
12. Device Deficiencies and Malfunctions 26
13. Ethical and Regulatory Considerations 27
13.1. Compliance with Good Clinical Research Practice 27
13.4. Protocol Compliance 28
13.6. Study Monitoring 28
13.7. Safety Reporting 30
13.8. Electronic Case Report Forms/Electronic Data Capture 31
13.9. Quality Assurance Audits 32
13.10. Records Retention 32
13.11. Publication and Reporting of Study Results 33
14. Personnel Responsibilities 33
14.1. Investigator responsibilities: 33
14.2. Sponsor Responsibilities 34

CONFIDENTIAL
All information contained in this document is confidential and is the sole property of Noctrix Health, Inc.
Any reproduction in part or whole without the written permission of Noctrix Health, Inc. is prohibited.

15. Investigator Qualifications 34
16. Study Procedures 35
17. Study Endpoints and Analysis Overview 48
18. Remote procedures 52
19. References 53

Study Design Overview Synopsis

**Title**	Open-label Extension Study to Evaluate Longer-Duration Response to the NTX100 Neuromodulation System for Patients with Medication-Refractory Primary Restless Legs Syndrome (RLS)
**Study Device**	NTX100 Neuromodulation System ("NTX100"), an investigational device developed by Noctrix Health, Inc. NTX100 is an external (non-implantable) neurostimulation device that is worn bilaterally on the lower legs.<br>Subjects are instructed to self-administer stimulation on each night that RLS symptoms are present, as needed, to reduce and relieve RLS symptoms. Multiple stimulation sessions may be applied with a maximum of 120min per night.
**Study Objective**	To assess longer-duration efficacy, tolerability, and adherence for patients with moderate to severe medication-refractory RLS with the NTX100 Neuromodulation System (NTX100).
**Study Population**	This study will enroll adults with primary moderate-severe medication-refractory RLS who previously completed the RESTFUL Study (Advarra IRB Protocol #Pro00050412).
**Study Design**	**Arm 1** (Direct Roll-Over Extension) – For subjects who successfully complete The RESTFUL Study at or after the initiation of CT-05 at a given clinical site. Arm 1 involves a 24-wk extension period of NTX100 device use followed by an 8-wk period without device use.<br>**Arm 2** (Control Group) – For subjects who successfully complete the RESTFUL Study prior to the initiation of CT-05 at a given site or for subjects who choose not to enroll into Arm 1. Subjects enrolled in Arm 2 will not receive treatment with the NTX100 device. Arm 2 involves follow-up phone calls to access RLS progression and medication usage at 8-wks, 16-wks, and 24-wks from the last date of RESTFUL study participation.<br>**Arm 3** (Delayed Extension) – For subjects who successfully complete the RESTFUL Study prior to the initiation of CT-05 at a given clinical site. Arm 3 involves a 24-wk extension period of NTX100 device use followed by an 8-wk period without device use. The sponsor will notify each site once Arm 3 is open to enroll study subjects for that site.

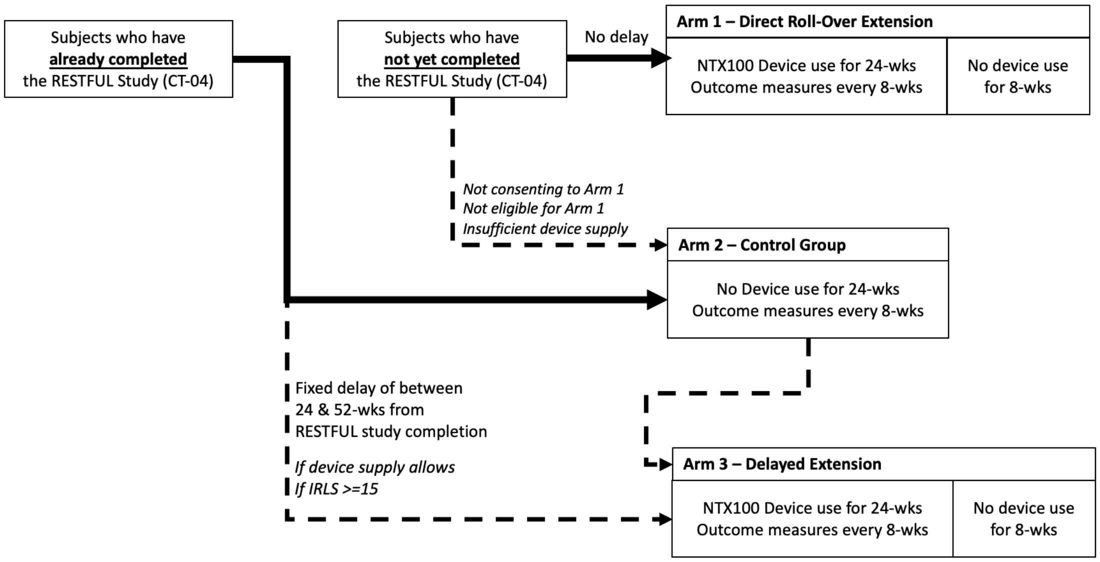

**Study Duration**	Arm 1: 32-wks, including 24-wk treatment duration and 8 weeks post study follow-up<br>Arm 2: 24-wks, with no treatment<br>Arm 3: 32-wks, including 24-wk treatment duration and 8 weeks post study follow-up
**Maximum Study Duration**	Arms 1 and 2 are expected to be completed within 12 months after the study opens to enrollment. Arm 3, if initiated, is expected to be completed within 24 months after the study opens to enrollment.
**Planned enrollment:**	Up to 132 subjects will be enrolled; all subjects who participated in the RESTFUL Study may be eligible. The same subject may be enrolled into both Arms 2 and 3; if so, this subject will count only once towards the total of 132.
**Number of Clinical Sites**	Up to 7 clinical sites within the United States, all of which participated in the RESTFUL Study.
**Randomization & Blinding**	There will be no randomization or blinding.<br>If interest exceeds investigational device availability for Arms 1 or 3, eligible subjects at each site will be recruited in the order that they completed The RESTFUL Study.
**Primary Efficacy Endpoint**	For Arm 1, the primary outcome measure will be responder rate on the CGI-I scale at Week 24 of Arm 1 relative to entry to the RESTFUL Study.<br><br>For Arm 3, the primary outcome measure will be responder rate on the CGI-I scale at Week 24 of Arm 3 relative to CT-05 study entry.

CONFIDENTIAL
All information contained in this document is confidential and is the sole property of Noctrix Health, Inc.
Any reproduction in part or whole without the written permission of Noctrix Health, Inc. is prohibited.

	Responder rate for the 7-point CGI-I scale will be defined as the proportion of responses of "Much Improved" or "Very Much Improved".
**Key Secondary Efficacy Endpoints**	1. PGI-I responder rate (defined as for CGI-I) at Week 24 of Arm 1 relative to entry to The RESTFUL Study.<br>2. Mean reduction in IRLS score at Week 24 of Arm 1 relative to entry to The RESTFUL Study.<br>3. Mean reduction in MOS-II score at Week 24 of Arm 1 relative to entry to The RESTFUL Study.<br>4. Mean reduction in MOS-I score at Week 24 of Arm 1 relative to entry to The RESTFUL Study.<br>5. Frequency of RLS symptoms (based on IRLS question #7) at Week 24 of Arm 1 relative to entry to The RESTFUL Study.
**Safety Endpoint**	Descriptive analysis of adverse events, classified and tabulated by seriousness, relationship to the device, timing, and severity.
**Adherence outcome measure**	Ratio of number of days of device use to number of days with RLS symptoms.
**Summary of subjective assessments**	CGI-I, PGI-I, IRLS, MOS-Sleep, SF-36, Weekly questionnaire, Bi-weekly questionnaire, Concomitant medications

Abbreviations

AE	Adverse Event
AO	Anticipated Observation
CFR	Code of Federal Regulations
CGI-I	Clinical Global Impressions – Improvement
CRF	Case Report Form
DCF	Data Clarification Form
EDC	Electronic Data Capture
FDA	Food and Drug Administration
GCP	Good Clinical Practice
IRB	Institutional Review Board
IRLS	International Restless Legs Syndrome Study Group Rating Scale
IRLSS	International Restless Legs Syndrome Society

CONFIDENTIAL
All information contained in this document is confidential and is the sole property of Noctrix Health, Inc.
Any reproduction in part or whole without the written permission of Noctrix Health, Inc. is prohibited.

ISO	International Organization for Standardization
MOS	Medical Outcomes Study
NPNS	Non-invasive peripheral nerve stimulation
NSR	Non-Significant Risk
PGI-I	Patient Global Impressions – Improvement
QA	Quality Assurance
RLS	Restless Legs Syndrome
SAE	Serious Adverse Event
SF-36	36-Item Short Form Health Survey
TENS	Transcutaneous Electrical Nerve Stimulation
USADE	Unanticipated Serious Adverse Device Effect

1. Background

A clinical need has been identified of improved treatment for those suffering with primary idiopathic restless legs syndrome (RLS). Patients with RLS have a strong urge with sensations of tingling/pain, usually in their legs, and often present with a primary complaint of not being able to fall asleep regularly. This leads to significant quality of life degradation, depression, daytime sleepiness, lack of productivity and a host of downstream effects associated with lack of quality sleep.

1.1. Restless leg syndrome: Background

Restless legs syndrome (RLS) is a sensorimotor disorder that is characterized by a distressing urge to move the legs and in some cases, other parts of the body such as arms [1]. The diagnosis is made by a response to five hallmark identifying criteria instituted by the International Restless Legs Syndrome Society (IRLSS) [2], as quoted below:

> "1. An urge to move the legs usually but not always accompanied by or felt to be caused by uncomfortable and unpleasant sensations in the legs.
>
> 2. The urge to move the legs and any accompanying unpleasant sensations begin or worsen during periods of rest or inactivity such as lying down or sitting.

All information contained in this document is confidential and is the sole property of Noctrix Health, Inc.
Any reproduction in part or whole without the written permission of Noctrix Health, Inc. is prohibited.

3. The urge to move the legs and any accompanying unpleasant sensations are partially or totally relieved by movement, such as walking or stretching, at least as long as the activity continues.

4. The urge to move the legs and any accompanying unpleasant sensations during rest or inactivity only occur or are worse in the evening or night than during the day.

5. The occurrence of the above features are not solely accounted for as symptoms primary to another medical or a behavioral condition (e.g., myalgia, venous stasis, leg edema, arthritis, leg cramps, positional discomfort, habitual foot tapping)."

Diagnostically, RLS is considered either primary, often occurring within families, or secondary, developing in association with other conditions (such as iron deficiency anemia, pregnancy or end-stage renal disease).

1.2. Epidemiology

In the United States, RLS is believed to affect more than 10 million adults and an estimated 1.5 million children and adolescents [3]. About one-third of those with RLS symptoms are bothered sufficiently enough to seek medical attention. Epidemiologic studies also show that women are at least 50% more susceptible to RLS than men and RLS is more common in older adults, although it can occur in some as early as the pre-school years.

1.3. Clinical treatments available

The current standard of care involves initial prescription of dopaminergic medications – such as Requip, Mirapex, and Neupro – which initially provide symptomatic relief but often become ineffective over continued usage [4]. Tolerance to these medications is rapid and well-documented [5]; approximately 10% of patients per year become refractory to these medications, and fewer than 20% patients have sustained benefits lasting 10 years or longer [6]. It is also now understood that dopaminergic medications cause what is known as "augmentation", or paradoxical progressive worsening of RLS symptoms that is much faster than the natural progression of the condition. Due to augmentation, patients on dopaminergic medications require increasingly higher doses [7]. Maximal dosage is limited by an increasing risk of side-effects at higher doses, which include compulsive behaviors including substance abuse, hypersexuality, and gambling [8]. As a result of these downsides of dopaminergic agents, a minority of clinicians are starting to prescribe gabapentinioids (e.g. Horizant) as an alternative first-line of treatment; these medications do not typically lead to

CONFIDENTIAL
All information contained in this document is confidential and is the sole property of Noctrix Health, Inc.
Any reproduction in part or whole without the written permission of Noctrix Health, Inc. is prohibited.

augmentation but confer risks such as respiratory depression [9], dizziness, and somnolence during the day.

For the large subpopulation of patients who become refractory to dopaminergic medications – typically due to augmentation – there are no FDA approved treatment options and no safe treatment options. As a result of tolerance, augmentation, and dosage limitations, RLS patients often continue to suffer from moderate-severe RLS symptoms while continuing to be reliant on high doses of dopaminergic medications to provide a small degree of relief. To address the massive unmet need, the leading clinicians involved with RLS advocate prescribing off-label opioids [10]. The leading options – oxycodone and methadone, have well documented risks, which include addiction, dependence, overdose, and occasionally death. This situation is especially concerning because primary RLS typically starts in middle age or earlier and persists throughout life, thus patients may end up reliant on opioids for the final decades of their lives.

1.4. Investigational Procedure

The investigational device – the NTX100 Neuromodulation System – is a non-invasive nerve peripheral stimulation (NPNS) device developed by Noctrix Health, Inc. (Sponsor) and designed to bilaterally stimulate the common peroneal nerve. Stimulation electrodes are positioned superficially and bilaterally on the lower legs over the head of the fibula bone, a position where the peroneal nerve is closest to the skin. This nerve target innervates regions of the lower extremities commonly associated with RLS symptoms.

1.5. Rationale

The NTX100 Neuromodulation System is a NPNS device that is inspired by the temporary symptomatic relief that RLS patients experience during voluntary movement of their lower extremities. One of the fundamental diagnostic criteria for RLS is the fact that patients report short-lived symptomatic relief during voluntary movement of their legs and feet [2]. This is conceptually similar to the gate-control theory mechanism of pain relief, wherein activation of large sensory fibers suppresses pain signals. However, for RLS, it appears that muscle activation is especially effective – activating sensory fibers

CONFIDENTIAL
All information contained in this document is confidential and is the sole property of Noctrix Health, Inc.
Any reproduction in part or whole without the written permission of Noctrix Health, Inc. is prohibited.

of the affected region of the body (e.g. via rubbing or touching) does not typically result in similar relief.

Non-invasive electrical stimulation is known to elicit muscle activation – this feature is used by two types of approved medical devices – powered muscle stimulators (21 CFR 890.5850) and external functional neuromuscular stimulators (21 CFR 882.5810). However, these devices tend to have distracting side-effects including paresthesia and phasic muscle twitches, and thus are incompatible with sleep and inappropriate for use in treating a sleep condition such as RLS. In contrast, the NPNS investigational device tested here produces waveforms that are designed to generate muscle activation without these paresthesia and phasic muscle twitches. Therefore, this technology may be compatible with sleep.

The NTX100 Neuromodulation System stimulates the peroneal nerve at its most superficial position over to the head of the fibula bone to activate muscles of the lower leg including the tibialis anterior at intensity levels that are comfortable and non-distracting. Such comfortable tonic muscle activation is designed to suppress RLS symptoms in a manner similar to voluntary movement – but unlike voluntary movement, may be compatible with sleep. Moreover, the output waveforms and intensities of the NTX100 Neuromodulation System are similar to Functional Electrical Neuromuscular Stimulators and Transcutaneous Electric Nerve Stimulation (TENS) Devices for treatment of pain, two classes of devices designated nonsignificant risk per 21 CFR 812.3. Therefore, there is a reasonable expectation that the NTX100 Neuromodulation System will lack significant risks.

This study evaluates the effects of NPNS on the symptoms of RLS during in-home subject-administered stimulation. This approach is useful for evaluating safety, usability, tolerability, and preliminary efficacy in a realistic environment - thus identifying any and all barriers to effective and tolerable use.

1.6. The RESTFUL Study

The Sponsor of this study previously received IRB approval for "A Multi-Center, Randomized, Double-Blind, Sham-Controlled Study to Evaluate the NTX100 Neuromodulation System for Patients with Medication-Refractory Primary Restless Legs Syndrome (RLS) – The RESTFUL study"

(Advarra IRB Protocol #Pro00050412), which has the objective of providing comparative evidence assessing clinically meaningful benefit in the treatment of patients with moderate to severe

All information contained in this document is confidential and is the sole property of Noctrix Health, Inc.
Any reproduction in part or whole without the written permission of Noctrix Health, Inc. is prohibited.

medication-refractory RLS with the NTX100 Neuromodulation System. The enrollment for "The RESTFUL study" began in May 2021, and is currently enrolling subjects as of November 2021. The current study is intended as an extension to the RESTFUL Study and thus is limited to subjects who previously completed the RESTFUL Study.

2. Regulatory Status

The Sponsor has determined that the investigational device in this study – the NTX100 Neuromodulation System – is a nonsignificant risk device under 21 CFR §812.2(b). In previously approved investigational protocols RLS-SNS01 and CT-03, Western IRB confirmed that earlier NTX100 Neuromodulation System prototypes - with comparable stimulation output - were nonsignificant risk devices. The investigational protocol CT-04 uses the same NTX100 Neuromodulation System prototype planned for use in this study, which was also approved as an nonsignificant risk device study by Advarra IRB on March 25, 2021. Therefore, an approved Investigational Device Exemption (IDE) from FDA is not required to legally perform the study described herein in the US.

The rationale for the nonsignificant risk determination is that the NTX100 Neuromodulation System has equivalent risks and comparable electrical stimulation parameters to Transcutaneous Electric Nerve Stimulation (TENS) Devices for treatment of pain (except for chest pain/angina)," which are Nonsignificant risk (NSR) device per the FDA guidance on Significant Risk and Nonsignificant Risk Medical Device Studies [11].

3. Objective

The study objective is to assess longer-duration efficacy, tolerability, and adherence for patients with moderate to severe medication-refractory RLS with the NTX100 Neuromodulation System (NTX100).

4. Study Design

Subjects who have previously completed The RESTFUL Study are consented to one of three Arms, the design of which is illustrated in the flowchart below (Figure 1). Arm 1 is a Direct Roll-Over Extension, Arm 2 is a Control, and Arm 3 is a Delayed Extension. A subject may be asked to participate in Arm 3 after completing Arm 2.

- **Arm 1** (Direct Roll-Over Extension) – Arm 1 is only an option for subjects who successfully complete The RESTFUL Study after enrollment to CT-05 begins at a given clinical site; Arm 1 involves a 24-wk extension period of NTX100 device use followed by an 8-wk period without device use.
- **Arm 2** (Control Group) – Arm 2 is an option for subjects who successfully complete the RESTFUL Study before enrollment to CT-05 begins at a given clinical site or subjects who decline to participate in Arm 1. Subjects enrolled in Arm 2 will not receive treatment with the NTX100 device. Arm 2 involves follow-up phone calls to access on RLS progression and medication usage at 8-wks, 16-wks, and 24-wks after the end of The RESTFUL Study. Subjects who are consented more than 8-wks after The RESTFUL Study completion will only complete the remaining follow-ups (e.g. subjects consented 12-wks after The RESTFUL Study will only complete the 16-wk and 24-wk follow-ups).
- **Arm 3** (Delayed Extension) – Arm 3 is only an option for subjects who successfully complete The RESTFUL Study before enrollment to CT-05 begins at a given clinical site; like Arm 1, Arm 3 involves a 24-wk extension period of NTX100 device use followed by an 8-wk period without device use. Enrollment in Arm 3 of the study begins after a fixed delay upon close-out of the RESTFUL Study; the fixed delay will be >= 24-wks and <= 52-wks, will be the same value for all subjects (+/- 2-wks), and will be determined based on investigational device availability and other constraints. The sponsor will notify each site once Arm 3 is open to enroll study subjects for that site.

.

CONFIDENTIAL
All information contained in this document is confidential and is the sole property of Noctrix Health, Inc.
Any reproduction in part or whole without the written permission of Noctrix Health, Inc. is prohibited.

Figure 1: Study Design Flowchart

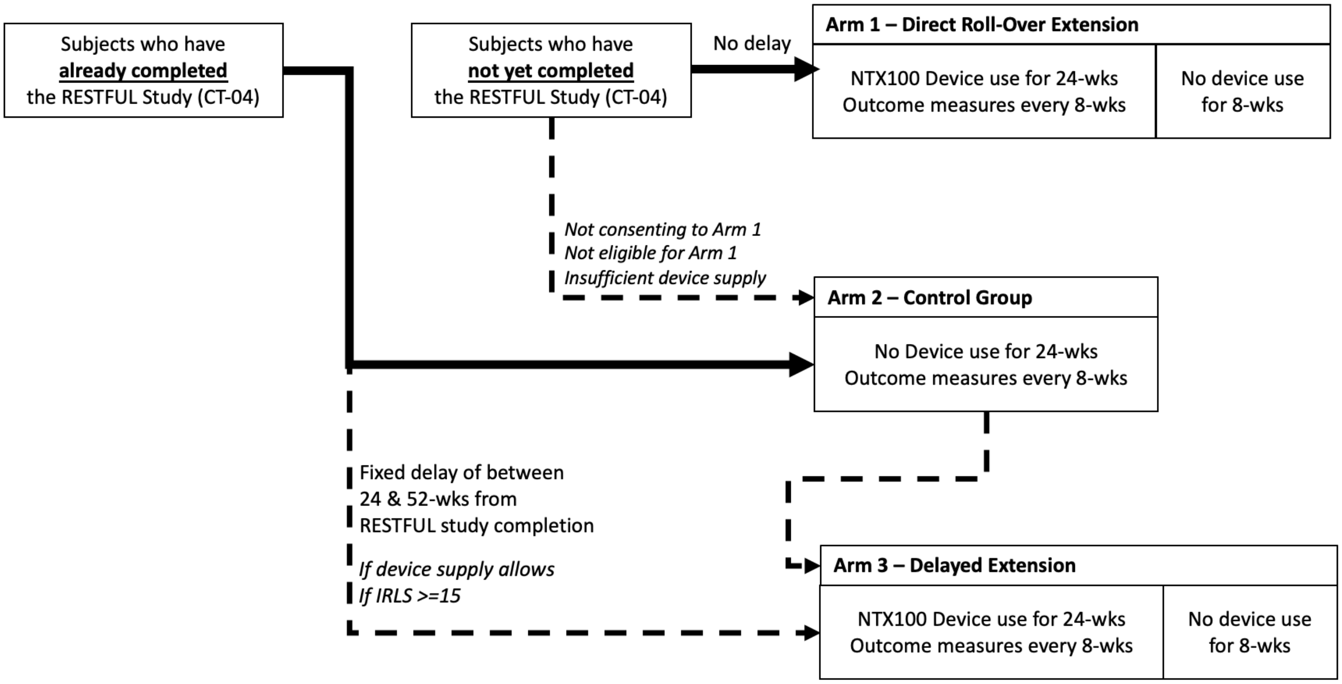

5. Blinding

Arms 1 and 3 will be open label. The treatment assignment on each device will be set to Active for all subjects and subjects will be informed that they are received Active treatment.

Arm 2 does not involve treatment.

6. Duration

The study will be completed approximately 18 months after the study opens to accrual.

7. Study population overview

The study population will consist of adults with moderate-severe primary RLS who have previously completed The RESTFUL Study. The following inclusion and exclusion criteria are designed to reduce confounding variables and reduce risk.

7.1. Screening process differences for each Arm

All subjects were screened for the eligibility criteria in Sections 7.2 and 7.3 upon entry to The RESTFUL Study, with the exception of IC #13 and EC #25-27. The following subset of eligibility criteria will be re-assessed at screening for CT-05, to confirm that they still apply:

- **Arm 1:** IC #8, 12-13. EC #7, 11*, 12, 14-17, 19, 21-27.
  ** except that acute infections with mild symptoms will not be exclusionary.*
- **Arm 2:** IC #12-13. EC #12, 17, 21, 23, 25.
- **Arm 3:** IC #3-8, 10, 12-13. EC #2, 3, 6-12, 14-17, 19-27.

7.2. Complete list of inclusion criteria (IC)

All subjects are required to meet the following inclusion criteria in order to be considered eligible for participation in this study:

1. Subject has received a medical diagnosis of primary restless legs syndrome (RLS).
2. Subject is refractory to RLS medication (as defined in section 7.3).
3. Subject has moderate-severe RLS symptoms as defined by a score of 15 or greater points on IRLS (International Restless Legs Syndrome Study Group Rating Scale [12]) over the week prior to study entry.
4. Subject has RLS symptoms 2 or more nights per week during the week prior to study entry as defined by a score of 2, 3, or 4 on IRLS question #7.
5. RLS symptoms are most significant in the subject's lower legs and/or feet.
6. RLS symptoms are most significant at bedtime, after bedtime, and/or in the 2 hours before bedtime.
7. RLS symptoms between 10am and 6pm are not severe.
8. Subject agrees to not change dosage or schedule of any medications that are known to impact RLS symptoms during the study, including RLS medications, antidepressants, sleep medications, or sedative antihistamines.
9. Subject agrees to not make major lifestyle changes during the study that would significantly affect bedtime, such as major changes to diet, exercise, or career.

10. Subject possesses the necessary equipment, internet/phone accessibility, and communication ability to complete electronic questionnaires and respond to electronic communications and phone calls from the research staff throughout the in-home portion of the study.
11. Subject is ≥ 22 and ≤ 79 years of age when written informed consent is obtained.
12. Subject has signed a valid, IRB-approved informed consent form, can understand the requirements of the study and instructions for device usage, and can converse in English.
13. Subject previously completed the RESTFUL Study.

7.3. Complete list of exclusion criteria (EC)

Subjects will be excluded from participating in this study if they meet any of the following exclusion criteria:

1. Subject has RLS that is known to be caused by another diagnosed condition (i.e. secondary RLS).
2. Subject is taking an unstable or inconsistent dose or schedule of medication that is likely to impact RLS symptoms, such as antidepressants, sleep medications, or sedative antihistamines or has changed dosage within the past 30 days.
3. Subject has changed dose and schedule of RLS medications within the month prior to study entry or is otherwise on an inconsistent dose or schedule of RLS medications.
4. Subject has prior experience with neurostimulation devices developed by the study sponsor, has prior experience using neurostimulation devices to treat RLS symptoms, or intends to use a neurostimulation device other than the study device during the study period.
5. Subject was misdiagnosed with RLS, as determined by the investigator (e.g. actual diagnosis of PLMD, arthritis, leg spasms or neuropathy without comorbid RLS).
6. Subject has a primary sleep disorder other than RLS that significantly interferes with sleep at the present time (e.g. obstructive sleep apnea stably controlled via CPAP would not be an exclusion).
7. Subject has active medical device implant anywhere in the body (including but not limited to pacemakers, spinal cord stimulators, deep brain stimulators) or metal implant at the site of study device electrode application.
8. Subject has severe peripheral neuropathy affecting the lower legs and/or subject has neuropathy and is unable to clearly distinguish between symptoms of neuropathy and symptoms of RLS.

CONFIDENTIAL
All information contained in this document is confidential and is the sole property of Noctrix Health, Inc.
Any reproduction in part or whole without the written permission of Noctrix Health, Inc. is prohibited.

9. Subject reports that bedtime is typically outside of 9pm-3am or reports that bedtime regularly varies by more than 4 hours, such as due to shift work.
10. On nights with no RLS symptoms (if any), subject reports typical sleep onset latency of >60min.
11. Subject has been diagnosed with one of the following conditions:
    - Epilepsy or other seizure disorder
    - Current, active or acute or chronic infection other than common cold
    - A malignancy within the past 5 years (not including basal or squamous cell skin cancer)
    - Stage 4-5 chronic kidney disease or renal failure
    - Severe movement disorder symptoms (Parkinson's disease, Huntington's disease, dyskinesia, dystonia)
    - Deep vein thrombosis
    - Multiple sclerosis
12. Subject has moderate or severe cognitive disorder or mental illness.
13. Subject has current diagnosis of iron-deficient anemia or history of iron-deficient anemia within the past year.
14. Subject has known allergy to device materials, electrode gel, polyurethane foam, or lycra (or severe previous reaction to medical adhesives or bandages).
15. Subject has severe edema affecting lower legs.
16. Subject has any of the following at or near the location of device application.
    - Acute injury
    - Cellulitis
    - Open sores
    - Other skin condition
17. Subject is on dialysis or anticipated to start dialysis while participating in the study.
18. During the NTX100 calibration process, which is identical for subjects in the active and sham arms, subject reports not feeling stimulation sensations up to an intensity of 30mA, the subject finds stimulation intensities less than 15 mA to be uncomfortable or distracting, or the device does not properly fit the subject.

CONFIDENTIAL
All information contained in this document is confidential and is the sole property of Noctrix Health, Inc.
Any reproduction in part or whole without the written permission of Noctrix Health, Inc. is prohibited.

19. Subject has received another investigational device or drug within 30 days before study entry, is planning to receive another investigational device or drug during the study, or is planning to change RLS medications during the study.
20. Subject has undergone a major surgery (excluding dental work) in the 30 days prior to study entry.
21. Subject is unable or unwilling to comply with study requirements.
22. Subject is pregnant or trying to become pregnant.
23. Subject has a medical condition not listed above that may affect validity of the study as determined by the investigator.
24. Subject has a medical condition not listed above that may put the subject at risk as determined by the investigator.
25. Subject was unwilling or unable to follow instructions in the RESTFUL Study, including missing 2 or more follow-up evaluations.
26. Subject failed to properly operate the investigational devices during Weeks 5-8 of the RESTFUL Study.
27. Subject experienced a significantly higher than expected rate of device malfunctions in the RESTFUL Study, such that the malfunctions appeared to be directly related to the subject's pattern of use.

7.4. Definition of RLS Refractory to Medication (IC #2)

For inclusion criterion #2, the following definition of refractory will be used:

The patient has failed at least one prescription medication administered to treat RLS* for one or more of the following reasons, as determined by the investigator:

1. Adverse effects associated with the medication are intolerable.
2. Patient exhibits symptoms of augmentation.
3. Efficacy has reduced to the point where an up titration would be needed to maintain a sufficient response to medication.

CONFIDENTIAL
All information contained in this document is confidential and is the sole property of Noctrix Health, Inc.
Any reproduction in part or whole without the written permission of Noctrix Health, Inc. is prohibited.

4. The patient lacks sufficient response to medication at the maximum approved or recommended dosage.**

5. The patient lacks sufficient response to medication at the maximum tolerable dosage due to adverse effects.

**Failure of any of the following prescription medications is sufficient to demonstrate that a patient is refractory: Ropinirole (FDA-approved), Pramipexole (FDA-approved), Rotigotine (FDA-approved), Gabapentin enacarbil (FDA-approved), Pregabalin (not FDA-approved for RLS, but administered in clinical practice), Gabapentin (not FDA-approved for RLS, but administered in clinical practice).*

*** per FDA-approved dosage regimen or per RLS consensus statement in the case of off-label medications (Pregabalin or Gabapentin).*

8. Investigational Device

8.1. Description

The NTX100 Neuromodulation System is positioned and worn bilaterally on the legs with stimulation electrodes over the head of the fibula bone, thus targeting the common peroneal nerve – this nerve target innervates regions of the lower extremities commonly associated with RLS symptoms.

For each leg, the NTX100 Neuromodulation System consists of (1) one stimulation unit, (2) two or more electrode areas, and (3) a mechanism for attachment to leg, described below.

- The (1) stimulation unit will:
  - be battery-powered and contain a rechargeable battery and connector for recharging,
  - contain a circuit board that generates the stimulation waveform,
  - contain controls that the subject can use to activate stimulation,
  - contain controls that the subject can use to adjust the intensity of stimulation within a range programmed by the researcher,
  - contain an electronic connection mechanism to deliver a stimulation waveform to the electrodes,

  - contain an interface that the researcher can use to program the range of stimulation intensities, program the mode of stimulation (active or sham), and download data on compliance and functionality.
- The (2) electrodes areas will each:
  - be an electrolyte-containing medium or a hydrogel material,
  - be disposable, allowing for at least 1 night of use by one subject,
  - be attached to the stimulation device by using snaps, magnetic connectors, conductive adhesives, shielded or insulated wires, hook and loop fasteners, or similarly secure connectors,
  - have an electrode surface area between 5cm2 and 50cm2.
- The (3) attachment mechanism will:
  - secure the device containing the disposable electrode areas to the body via straps and/or additional biocompatible adhesives.

Description of stimulation parameter ranges allowed by protocol:

- Pulse amplitude: up to 45mA
- Frequency: 2kHz - 6kHz
- Pulse width: 80 - 250 microseconds (depending on frequency)
- Duty cycle: 25-100%
- Pulse shape: Charge-balanced
- Duration: up to 60 minutes per session (up to 120 minutes per day)

8.2. Instructions for Use and Administration

Use of the NTX100 Investigational device is described briefly herein. For details see Instructions for Use.

8.2.1. Route of administration

The NTX100 Neuromodulation System is designed to stimulate the common peroneal nerve; stimulation electrodes within the device are positioned superficially and bilaterally on the lower

All information contained in this document is confidential and is the sole property of Noctrix Health, Inc.
Any reproduction in part or whole without the written permission of Noctrix Health, Inc. is prohibited.

legs over the head of the fibula bone, a position where the peroneal nerve is close to the skin. This nerve target innervates regions of the lower extremities commonly associated with RLS symptoms.

8.2.2. Dosage and dosage regimen

The dosage regimen depends on the intensity, timing, and duration of stimulation:

1. Intensity. The setpoint stimulation intensity may be the lesser of (a) the maximal intensity that the subject reports is non-distracting and comfortable, as determined in the calibration session described in the instructions for device use and (b) the maximal intensity for each set of stimulation parameters, as determined based on device output capabilities. Subjects may be allowed to adjust stimulation intensity from the setpoint stimulation intensity, but only within a limited pre-programmed range. For example, the subject may increase intensity if symptoms are severe or reduce intensity if symptoms are mild.

2. Timing. Subjects may be instructed to administer stimulation primarily after RLS symptoms become noticeable and before the RLS symptoms become severe.

3. Duration. Each session of stimulation may last 30 minutes. Stimulation may be activated for up to 120 minutes per day (e.g., up to 4 x 30-minute sessions), depending on the timing, duration, and severity of RLS symptoms for the specific subject.

8.3. Contraindications

The following are contraindications to device usage:

- Diagnosis of epilepsy or other seizure disorder,
- Active medical device implant anywhere in the body, including but not limited to pacemakers, spinal cord stimulators, deep brain stimulators
- Metal implant at the site of study device application
- Known allergy to device materials (or severe previous reaction to medical adhesives or bandages)
- Cellulitis, open sores, or acute injury at or near the location of device electrode application

CONFIDENTIAL
All information contained in this document is confidential and is the sole property of Noctrix Health, Inc.
Any reproduction in part or whole without the written permission of Noctrix Health, Inc. is prohibited.

9. Risks

9.1. Investigational device risks

NPNS devices such as NTX100 Neuromodulation System result in a known potential for the following anticipated observations, which are typically mild to moderate, transient in nature, and resolve over time.

- Mild skin irritation from use of adhesive electrodes and/or secondary attachment mechanism.
- Discomfort, paresthesia, or otherwise irritating or uncomfortable sensations during active electrical stimulation.
  - *This risk is reduced by calibrating the stimulation intensity.*
- Temporary interference with sleep while wearing the device:
  - For some individuals, device may be uncomfortable, thus interfering with sleep while wearing the device.
  - For some individuals, this device may interfere with preferred sleep positions, thus interfering with sleep during usage.
- Temporary increase in RLS symptoms while wearing the device:
  - For some individuals, this device may interfere with voluntary leg movements used to relieve RLS symptoms, thus leading to a temporary increase in RLS symptoms.
  - In some cases, this device may otherwise lead to a temporary increase in RLS symptoms during active stimulation for other reasons.
    - *This risk may be reduced by calibrating the stimulation intensity and/or adjusting the schedule of stimulation.*

10. Adverse Events

Anticipated observations (e.g. common treatment effects) and adverse events occurring during the study will be recorded. Descriptions of anticipated observations and AEs will include the date of onset, the date it ended, the severity, the relationship to study device, and the outcome. All reported AEs

CONFIDENTIAL
All information contained in this document is confidential and is the sole property of Noctrix Health, Inc.
Any reproduction in part or whole without the written permission of Noctrix Health, Inc. is prohibited.

will be summarized by the number of subjects reporting AEs, system organ class (where applicable), severity, seriousness, and relationship to study device.

The study Investigator and Coordinator will evaluate, characterize and record in the electronic Case Report Form (eCRF) all adverse events (AEs) occurring in all subjects from the time of enrollment to study exit (or premature withdrawal). Adverse events unresolved at study exit will be followed by the Investigator until resolution occurs or at least 30 days after the subject's participation in the study is complete. AEs may be reported spontaneously by the subject or detected by the Investigator or Coordinator. AEs should be evaluated for diagnoses not just symptoms (i.e., "angina", not "chest pain").

In addition to verbatim terms, the Sponsor may categorize all AEs using MedDRA preferred terms (PT) and system organ classes (SOC). Analysis may report both verbatim and MedDRA terms.

10.1. Adverse Event Definitions

An **adverse event** (AE) is any untoward medical occurrence, independent of its association with the investigational device. AEs also include any adverse laboratory signs or physical exam findings.

A **serious adverse event** (SAE[1]) is any AE that:

- led to a death,
- led to a serious deterioration in the health of the subject that:
  - resulted in a life-threatening illness or injury,
  - resulted in a permanent impairment of a body structure or a body function,
  - required in-patient hospitalization or prolongation of existing hospitalization,
  - resulted in medical or surgical intervention to prevent permanent impairment to body structure or a body function
- or led to fetal distress, fetal death or a congenital abnormality or birth defect.

A **device-related SAE** is an event meeting the SAE definition above that is also rated as probably or definitely related the investigational device. No device-related SAEs have been reported in prior

[1] Definition from ISO14155:2011

CONFIDENTIAL
All information contained in this document is confidential and is the sole property of Noctrix Health, Inc.
Any reproduction in part or whole without the written permission of Noctrix Health, Inc. is prohibited.

studies. Note that an elective or pre-planned hospitalization for a condition that did not worsen during the study is not an AE.

An **unanticipated serious adverse device effect** (USADE) is any SAE that is caused by, or associated with, a device, if that effect, problem, or death was not previously identified in nature, severity, or degree of incidence in the investigational plan or application (including a supplementary plan or application).

10.2. AE Severity and Relatedness

Each AE occurring in the study will be characterized by the study Investigator as to severity (Table 1) and relatedness (Table 2).

Table 1. AE Severity Grading System.

Grade	Brief description
Grade 1	Mild; asymptomatic or mild symptoms; clinical or diagnostic observations only; intervention not indicated
Grade 2	Moderate; minimal, local, or non-invasive intervention indicated; limiting age-appropriate instrumental ADL[a]
Grade 3	Severe or medically significant but not immediately life-threatening; hospitalization or prolongation of hospitalization indicated; disabling; limiting self-care ADL[b]
Grade 4	Life-threatening consequences; urgent intervention indicated
Grade 5	Death related to adverse event

[a]'Instrumental ADL' refers to activities of daily living such as preparing meals, shopping for groceries or clothes, using the telephone, and managing money. [b]'Self-care ADL' refers to bathing, dressing and undressing, feeding oneself, using the toilet, taking medications, and not being bedridden. From the National Cancer Institute Common Terminology Criteria for Adverse Events v4.0 NCI, NIH, DHHS. May 29, 2009 NIH publication #- 09-7473.

Table 2. AE Relatedness Grading System.*

Grade	Relationship of AE to study device	Description
5	Definite	An event that follows a reasonable temporal sequence from administration of the study device; that follows a known or expected response pattern to the study device; and that is confirmed by improvement on stopping.
4	Probable	An event that follows a reasonable temporal sequence from administration of the study device; that follows a known or expected response pattern to the study device; and that is unlikely to have been caused by concurrent/underlying illness or other drugs, procedures, or other causes.
3	Possible	An event that follows a reasonable temporal sequence from administration of the study device; that follows a known or expected response pattern to the study device; but may have been caused by concurrent/underlying illness, drugs, procedure, or other causes.
2	Unlikely	An event that does not follow a reasonable temporal sequence from administration of the study device; that does not follow a known or expected response pattern to the study device, or most likely was caused by concurrent/underlying illness, drugs, procedure, or other causes, because of their known effects.
1	Not related	An event almost certainly caused by concurrent/underlying illness, drugs, procedure, or other causes.

** AEs occurring before treatment with the study device will be categorized as unrelated to the study device.*

10.3. Adverse Event Reporting

Investigators must report all SAEs to the study Sponsor and governing IRB within 3 business days or according to local IRB guidelines. Investigators should call the study sponsor immediately upon becoming aware of the occurrence of an SAE. The sponsor will contact the independent medical reviewer, to assist in assessing any safety concerns, if needed. The Investigator should be able and willing to provide further information on the specific event when requested by the study Sponsor. If the Investigator learns of an SAE that occurs within 1 month after the subject completes the study,

CONFIDENTIAL
All information contained in this document is confidential and is the sole property of Noctrix Health, Inc.
Any reproduction in part or whole without the written permission of Noctrix Health, Inc. is prohibited.

he/she should notify the Sponsor. Investigators must also report all AEs to the governing IRB as determined by that IRB.

Prompt AE evaluation:

- protects the safety of study subjects;
- aids in understanding the overall safety profile of the device;
- prompts, if necessary, modification to the study protocol
- allows improvements in study design or procedures; and
- adheres with standard good clinical practices.

11. Device Tracking

The Sponsor will send the investigational devices to the Investigator. The Investigator must house study devices in a secure location.

The Investigator must carefully and completely track receipt, use and disposition of all investigational devices. The Sponsor will track sending and receiving of devices. The Sponsor will monitor site device accountability periodically.

If a Sponsor representative or designee is present at the time of use, he/she may directly take possession of used device(s). All devices will be returned to the Sponsor after the study is complete.

The investigational device may include components that are consumable or semi-consumable and may be replaced on a periodic or as-needed basis. Such device replacement can occur via an in-person exchange or via delivery to the subject via courier from the site.

12. Device Deficiencies and Malfunctions

Throughout the study, the Investigator and study staff will report and document all device deficiencies and malfunctions related to the identity, quality, durability, reliability, safety or performance of the device. This includes reporting of device deficiencies/malfunctions that did not lead to an AE but could have if: 1) suitable action had not been taken, 2) intervention had not been made, or 3) circumstances had been less fortunate. If possible, the Investigator should return devices suspected of deficiency or malfunction to the Sponsor for analysis.

CONFIDENTIAL
All information contained in this document is confidential and is the sole property of Noctrix Health, Inc.
Any reproduction in part or whole without the written permission of Noctrix Health, Inc. is prohibited.

13. Ethical and Regulatory Considerations

13.1. Compliance with Good Clinical Research Practice

This study will be conducted in compliance with the principles of the Belmont Report, the Declaration of Helsinki, with the current Good Clinical Practice (GCP) guidelines and with other applicable regulations. The Investigator and all study staff will conduct the study in compliance with this protocol. Voluntary informed consent will be given by every subject prior to the initiation of any study-related procedures. The rights, safety and well-being of the study subjects are the most important considerations and prevail over the interests of science and society. All personnel involved in the conduct of this study must be qualified by education, training and experience to perform their assigned responsibilities.

13.2. Confidentiality of Data

All information and data sent to the Sponsor, Contract Research Organizations, or the Independent Medical Reviewer concerning subjects or their participation in this study will be considered confidential. All data used in the analysis and reporting of this evaluation will be used in a manner without identifiable reference to the subject. The investigator consents to visits by the staff of the Sponsor and its authorized representatives and the U.S. Food and Drug Administration or any other governmental body to review the study subjects' medical records including any test or laboratory data.

13.3. Institutional Review Board (IRB) and Informed Consent

Before study initiation, the Investigator must have written and dated approval from the IRB for the protocol, consent form, subject recruitment materials/process (e.g., advertisements), and any other written information to be provided to subjects. The Investigator should also provide the IRB with a copy of the product labeling, information to be provided to subjects and any updates. The Investigator will submit documentation of the IRB approval to the Sponsor. Copies of all correspondence with the IRB regarding this study must be sent to the Sponsor.

CONFIDENTIAL
All information contained in this document is confidential and is the sole property of Noctrix Health, Inc.
Any reproduction in part or whole without the written permission of Noctrix Health, Inc. is prohibited.

The IRB-approved consent form must include all elements required by FDA, state, and local regulations, and may include appropriate additional elements.

The Investigator/designee will explain the study to each potential subject and the subject must indicate voluntary consent by signing and dating the approved informed consent form. The Investigator must provide the subject with a copy of the consent form in a language the subject understands. The Investigator will maintain documentation that informed consent was obtained prior to the initiation of any study-specific procedures.

Withdrawal of IRB approval of the Investigator's part in the investigation must be reported to the Sponsor within 5 working days.

13.4. Protocol Compliance

The Investigator will comply to the extent possible with the IRB-approved protocol. All deviations from the protocol must be documented. The Investigator will notify the Sponsor immediately if a deviation from the protocol was required to protect subject safety.

13.5. Protocol Revisions

Revisions to the study protocol can be made only by the study Sponsor. A revised protocol can be put into place only after governing IRB approval. All administrative letters must be submitted to the IRB for their information.

New or altered consent forms required by the IRB due to a protocol change must be signed by all subjects currently enrolled in the study and must be used for any subsequent subject enrollment.

13.6. Study Monitoring

Representatives of the Sponsor will conduct either on-site or virtual oversite visits for all study sites to perform monitoring and data management functions, and provide participating sites with relevant contact information, as necessary. Study monitors may change periodically over the

CONFIDENTIAL
All information contained in this document is confidential and is the sole property of Noctrix Health, Inc.
Any reproduction in part or whole without the written permission of Noctrix Health, Inc. is prohibited.

course of this study. All monitors will be qualified to perform their assigned responsibilities, and participating investigators/site personnel will be notified of any changes as they occur.

On-site and/or remote monitoring of all participating sites will be frequent enough to assure continued acceptability of the data by assessing site compliance with the study protocol, adherence to data collection procedures, and maintenance of study records. Scheduled site visits will include, but are not limited to, the following:

- Site initiation visit: prior to enrolling subjects, an on-site or remote initiation visit will be conducted by clinical study personnel to review this study protocol and discuss eCRF completion and transmittal procedures. Alternatively, a meeting may be conducted for several sites at a common location.

- Interim monitoring site visit: on-site and/or remote monitoring visits will be conducted at all sites to assess the progress of the study and identify any concerns that result from review of the study records, study management documents, or subject informed consent documents. To assure the integrity of the data, a representative number of individual subject records and other supporting documents will be compared to eCRFs completed at the site to determine that:

    - The study protocol is being followed, and only eligible subjects are being enrolled; variances, if they occur, are recorded and reported as appropriate.
    - Informed consent is properly documented.
    - Adverse events are being reported appropriately.
    - Information recorded on eCRFs is complete, accurate and legible.
    - Subjects failing to complete the clinical study and the reason for failure are properly recorded.

- Final monitoring/Close-out site visit: a final visit to participating sites may be made by the study monitor, if necessary. Any ongoing responsibilities will be discussed with the investigator and/or site personnel as appropriate.

CONFIDENTIAL
All information contained in this document is confidential and is the sole property of Noctrix Health, Inc.
Any reproduction in part or whole without the written permission of Noctrix Health, Inc. is prohibited.

At the close of the study at an investigational site, appropriately trained personnel appointed by the Sponsor will perform a close-out process via the telephone or on-site. The purpose of this visit is to collect all outstanding study data documents, ensure that the investigator's files are accurate and complete, review record retention requirements, ensure final accounting of all investigational devices shipped to the investigator, provide for appropriate disposition of any remaining supplies, and ensure that all applicable requirements are met for the study. The observations and actions made during the intervention will be documented and communicated to the investigator.

Representatives of government regulatory authorities may also evaluate the study records, source documents, Investigator, study staff and facilities.

The Investigator should immediately notify the Sponsor of any audits of this study by any regulatory agency, and must promptly provide copies of any audit reports.

13.7. Safety Reporting

The Sponsor is responsible for ongoing safety evaluation in this study protocol. Sponsor activities regarding safety include:

- classification of all AEs
- review of all AEs reported in the study
- confirm site's classification of AEs in terms of severity and relatedness to the study device
- review of severity and relatedness with the study Investigator, especially when there is disagreement between the Investigator and the sponsor
- review of device deficiencies and malfunctions, including determination and documentation of whether deficiencies/malfunctions could have led to an SAE
- ensuring the reporting of all SAEs and device deficiencies/malfunctions that could have led to an SAE to the IRB and, if required, regulatory authorities in a timely fashion
- informing all site Investigators in writing of all SAEs at all sites in a timely fashion
- updating the risk analysis and assessment of corrective or preventive actions potentially required as a result of new information obtained in the investigation

CONFIDENTIAL
All information contained in this document is confidential and is the sole property of Noctrix Health, Inc.
Any reproduction in part or whole without the written permission of Noctrix Health, Inc. is prohibited.

The Sponsor will evaluate all serious adverse events against US reporting requirements (Medical Device Reporting, 21 CFR 812) and Medical Device Directive (vigilance incident reporting) as per its standard operating procedures. The Sponsor will investigate each SAE to determine whether the event represents an unanticipated serious adverse device effect (USADE, see Section 7). The Sponsor will report any event to regulatory authorities, Investigators and reviewing IRBs/ECs as necessary. If an investigation shows that a USADE presents an unreasonable risk to subjects, the Sponsor will terminate all investigations or parts of investigations presenting that risk as soon as possible. The Sponsor will only resume a terminated investigation after corrective actions have taken place, site Investigators are informed and IRBs/ECs have been notified and given approval to resume the study.

13.8. Electronic Case Report Forms/Electronic Data Capture

The study will use an electronic data capture (EDC) system to implement electronic case report forms (eCRF). Data collected throughout the duration of the study will be entered directly into the EDC system. The system will allow compliance with 21 CFR 11 Electronic Signatures. All CRF's are housed in the EDC system. The Investigator and Coordinator will be trained in use of the eCRF prior to study initiation. Retraining in use of EDC can occur at any time. The EDC system will be validated prior to use and after any modification is made. Each eCRF will be designed to accommodate the specific features of the trial design. Modification of a eCRF will only be made if deemed necessary by the study sponsor.

An eCRF is required and should be completed for each included subject. The Investigator has ultimate responsibility for the collection and reporting of all data entered on the eCRFs and ensuring that they are accurate, authentic/original, attributable, complete, consistent, legible, timely (contemporaneous), enduring and available when required. The eCRFs must be signed by the investigator to attest that the data contained therein are true.

CONFIDENTIAL
All information contained in this document is confidential and is the sole property of Noctrix Health, Inc.
Any reproduction in part or whole without the written permission of Noctrix Health, Inc. is prohibited.

The site will be provided with general eCRF Completion Guidelines which will assist in data entry and data issues/questions. All persons allowed to enter or change eCRF data must appear on the Delegation of Responsibilities Log.

The Sponsor will remotely monitor the eCRFs to identify possible data errors. The system will have a query mechanism whereby the site Coordinator can respond to Sponsor queries. All data discrepancies will be resolved prior to database lock.

13.9. Quality Assurance Audits

Sponsor representatives or designees may conduct site quality assurance (QA) audits during the study. The Investigator must agree to provide the auditor with direct access to all relevant documents and discuss any findings with the auditor.

In the event of an inspection by the FDA or other regulatory authorities, the Investigator must give the inspector direct access to relevant documents and to discuss any findings with the inspector. The Investigator must notify Sponsor in the event of a FDA site audit.

13.10. Records Retention

The Investigator must maintain all study records (including device disposition, informed consents, supporting documents, correspondence, regulatory documents, contracts etc.) for at least 2 years after study completion. At the Investigator’s discretion, all records may be sent to the Sponsor for permanent storage.

The Investigator must contact the Sponsor or designee prior to destroying any records associated with this study. If the Investigator withdraws from the study, all study-associated records must be transferred to a mutually agreed upon designee. Written notification of such a transfer must be given to the Sponsor or designee.

CONFIDENTIAL
All information contained in this document is confidential and is the sole property of Noctrix Health, Inc.
Any reproduction in part or whole without the written permission of Noctrix Health, Inc. is prohibited.

13.11. Publication and Reporting of Study Results

The study will be registered with clinicaltrials.gov before the first subject is enrolled. Study results will be documented in a study report that will be signed by Sponsor representatives and by each Investigator who enrolls subjects in the study, unless otherwise noted.

If the results of this study will be published, all standard editorial and ethical practices, will be followed. Results from multi-center studies must be published or presented at congresses only in their entirety with data pooled from all centers. Individual Investigators may not publish data from individual centers, unless granted specific written permission from the Sponsor to do so.

The list of authors of any formal publication or presentation of study results may include, as appropriate, representatives of the Sponsor.

14. Personnel Responsibilities

14.1. Investigator responsibilities:

Investigators are responsible for ensuring the investigation is conducted according to all signed agreements, the study protocol, and applicable regulatory agency regulations (21 CFR 812), which include:

a) Permit monitor inspection of facilities and records.
b) Permit FDA and other government health authorities' inspection of facilities and records.
c) Submit protocol and informed consent to IRB and await approval.
d) Submit proposed amendments to protocol and informed consent to IRB and await approval, unless the change reduces the risk to subjects.
e) Obtain informed consent of subjects.
f) Implement study in accordance with protocol.
g) Complete case report forms.
h) Record and explain deviations from protocol and report to monitor.
i) Submit annual progress reports, final reports, and adverse effect reports to IRB and sponsor.

CONFIDENTIAL
All information contained in this document is confidential and is the sole property of Noctrix Health, Inc.
Any reproduction in part or whole without the written permission of Noctrix Health, Inc. is prohibited.

j) Record the receipt, disposition, and return of study devices.
k) Refrain from promoting study or study articles in such a way that the potential subject will be biased in his/her responses.
l) Maintain medical histories of subjects.
m) Retain records for two years following FDA approval of marketing application.

14.2. Sponsor Responsibilities

Listed below are the Sponsor's responsibilities for this study.

a) Assure IRB approval of protocol and informed consent is obtained.
b) Select and train monitors.
c) Select investigators.
d) Train investigators in device use.
e) Obtain Agreement Letter and curriculum vitae and proof of appropriate licensure of investigator and other study staff.
f) Control shipment of investigational devices.
g) Conduct day-to-day administration of study.
h) Investigate unanticipated, device related adverse effects.
i) Document protocol deviations and violations.
j) Obtain statement of financial disclosure.
k) Will appoint trained and qualified representative(s) to perform initial pre-screening.
l) May supervise device calibration.

15. Investigator Qualifications

Investigators must have an active license and board certification and experience in the treatment of RLS, as documented on their Curriculum Vitae and/or in a statement of the investigator's relevant experience, including dates, location, extent, and type of experience. Each Investigator must undergo training conducted by the Sponsor, on the study device prior to study initiation – device training conducted for the RESTFUL Study protocol will satisfy this requirement.

16. Study Procedures

16.1. Overview

Arms 1 and 3 involve a 24-week extension of in-home investigational device usage for subjects who previously completed the RESTFUL Study, followed by an 8-week period without investigational device usage to assess the progression of RLS following cessation of treatment. Two separate arms are employed to maximize the number of subjects eligible for any extension phase (Arm 1 and Arm 3) while also maximizing the number of subjects who can proceed directly to the extension phase (Arm 1 only). Notably, in the RESTFUL Study, subjects will have already completed either 4-weeks of device usage (Sham → Active) or 8-weeks (Active → Active) depending on randomization. Since site staff were blinded to RESTFUL Study treatment assignment, no procedures in this protocol will be affected by RESTFUL Study treatment assignment.

Arm 2 serves as a control arm with matched duration to the 24-week period of investigational device usage; this arm is designed to the assess the changes to medication and RLS symptoms that occur under the conventional standard of care, in the absence of investigational device intervention. Although the duration of Arm 2 states 24-weeks, each subjects participation will vary, since the 24-week period also includes the time between when the subject exits the RESTFUL study and when they enroll in Arm 2 of the CT-05 study. For example, subjects who are consented more than 8-wks after RESTFUL Study completion will only complete the remaining follow-ups (e.g. subjects consented 12-wks after the RESTFUL Study will only complete the 16-wk and 24-wk follow-ups).

16.2. Comparison of Study Arms

<u>Arms 1 and 3 involve identical procedures, with the following exceptions:</u>

- Eligibility
    a. [Arm 1] Only open to subjects who complete the RESTFUL Study <u>after</u> this protocol opens to enrollment at a given site - and thus can directly enter this protocol.

All information contained in this document is confidential and is the sole property of Noctrix Health, Inc.
Any reproduction in part or whole without the written permission of Noctrix Health, Inc. is prohibited.

b. [Arm 3] Only open to subjects who completed the RESTFUL Study before this protocol opens to enrollment at a given site - and thus have some delay before entering this protocol). Subjects who have completed Arm 2 remain eligible for Arm 3.

- Delay
  a. [Arm 1] There is typically no delay between RESTFUL Study completion and Arm 1 entry. A delay of up to 2-days is permitted if needed.
  b. [Arm 3] There is a fixed delay between RESTFUL Study completion and Arm 3 entry. The fixed delay will be >= 24-wks and <= 52-wks, will be the same value for all subjects (+/- 2-wks), and will be determined based on investigational device availability and other constraints.
- Baseline
  a. [Arm 1] Baseline refers to RESTFUL Study entry (Eval 1 of RESTFUL Study).
  b. [Arm 3] Baseline refers to entry to this protocol (Eval 1 of this protocol).
- Assessments at Eval 1
  a. [Arm 1] The IRLS and MOS-Sleep data from are not administered at Eval 1, because they are administered on the same day at Restful Study Eval 11.
  b. [Arm 3] The IRLS and MOS-Sleep are administered at Eval 1.

Arm 2 differs significantly from Arm 1 and 3:

- Eligibility
  a. [Cohort 2A] Subjects who completed the RESTFUL Study after this protocol opens to enrollment are eligible for Arm 2 only if they decide not to participate in Arm 1. All subjects in this group are first given the choice to participate in Arm 1 without knowledge of the alternative Arm 2.
  b. [Cohort 2B] All subjects who completed RESTFUL Study less than 24-wks before this protocol opens to enrollment are eligible for Arm 2.
- Delay

All information contained in this document is confidential and is the sole property of Noctrix Health, Inc.
Any reproduction in part or whole without the written permission of Noctrix Health, Inc. is prohibited.

a. [Cohort 2A] There is typically no delay between RESTFUL Study completion and Arm 2 entry. A delay of up to 2-days is permitted if needed.

b. [Cohort 2B] There is a variable delay between RESTFUL Study completion and Arm 2 entry, depending on the time that the subject completed the Restful Study. Subjects will be enrolled either 8-wks, 16-wks, or 24-wks after completion of the RESTFUL Study, +/- 1-wk.

- Completion

a. Both Cohorts 2A and 2B are completed 24-wks after completion of the RESTFUL Study +/- 1-week. Therefore, the duration of study participation in Arm 2 depends on the delay between RESTFUL Study completion and enrollment in this study.

- Baseline

a. For both Cohorts 2A and 2B, baseline refers to RESTFUL Study entry (Eval 1 of Restful Study).

Table 3. Comparison of Study Arms

	**Arm 1**	**Arm 2**	**Arm 3**
**Eligibility**	Later cohort of RESTFUL Study subjects	RESTFUL Study subjects who don't complete Arm 1	Earlier cohort of RESTFUL Study subjects
**Start date relative to RESTFUL Study completion**	0* days	Less than 24-wks	Fixed delay, >=24-wks and <=52-wks
**End date relative to RESTFUL Study completion**	32-wks	24-wks	Variable
**Duration**	32-wks	up to 24-wks	32-wks
**Investigational device use**	Wks 1-24	None	Wks 1-24
**Baseline**	RESTFUL Study entry	RESTFUL Study entry	Entry to this study

** Up to 2 days if needed*

16.3. Recruitment

Enrollment is limited to subjects who have completed the RESTFUL Study and meet the inclusion and exclusion criteria in Section 7. A pre-screening call may be completed to assess eligibility prior to the in-person visit and may be performed by a sponsor-appointed representative. Eligibility will be contingent on scheduling constraints, such as research facility and research staff availability.

16.3.1. Order of recruitment

For Arm 1, eligible subjects at a given site will be recruited in the order that they complete the RESTFUL Study, with the constraint that subjects will only be eligible if they complete the RESTFUL Study after enrollment to this study opens at that site.

For Arm 3, if enrollment or enrollment rate for a specific Arm and/or a specific site is limited – such as by device availability, research facility and research staff availability, or other constraints – then eligible subjects at a given site will be invited to this study in the order that they completed the RESTFUL Study.

These procedures are intended to minimize selection bias in recruitment. The actual order of enrollment for each Arm may differ depending on the responsiveness of each given subject that is invited and the ability of the subject and site to schedule Eval 1 for that subject in a timely manner.

16.4. Informed consent Process

All subjects must be provided a consent form describing the study with sufficient information for subjects to make an informed decision regarding their participation. The informed consent form will clearly indicate the Arm to which the subject is providing consent. If a given subject completes more than one Arm (e.g. Arm 2 followed by Arm 3), the subject will complete a separate consent form prior to enrollment in each Arm.

Subjects must sign the IRB approved informed consent prior to participation in any study specific procedure, with the exception of pre-screening. The subject must receive a paper or electronic copy of the signed and dated consent document. The signed copy of the consent document must be

CONFIDENTIAL
All information contained in this document is confidential and is the sole property of Noctrix Health, Inc.
Any reproduction in part or whole without the written permission of Noctrix Health, Inc. is prohibited.

retained in the study binder in paper or electronic format. The schedule of visits and procedures is provided in Table 4 (Arms 1 and 3) and Table 6 (Arm 2).

If a study site has a formal process for eConsent, they may elect to obtain informed consent virtually. Sites who do not currently have a process may elect to use the eConsent service provided by the EDC vendor.

16.5. Screening

Potential subjects will be screened for eligibility based on the criteria in Section 7 above. A potential subject who signs the study consent and is eligible will be assigned the same study ID number assigned to them during the RESTFUL Study, will be considered enrolled, and will count towards the study's sample size.

16.6. Device calibration

During the RESTFUL Study, the stimulation intensity of the investigational device was calibrated separately for each subject and for each leg. The calibration process is designed to identify the maximally effective settings that are comfortable for each subject. For Arm 1 and Arm 3, the same settings from the RESTFUL Study will be used, except in cases where the subject's responses indicate that these intensity levels are too high or too low. In that case, the calibration process may be repeated either in full or in an abbreviated manner. The sponsor may administer the calibration and/or assist in administering the calibration.

Each time new stimulation units are provided to a subject during this study, such as with replacement devices, they must be programmed with the specific calibrated settings corresponding to that subject.

16.7. Baseline Assessments

Since this is an extension study to the RESTFUL Study, many of the baseline assessments for a given subject in Arm 1 and Arm 2 will refer to assessments completed during the RESTFUL Study, including the following: IRLS [12], Subject characterization, Medical history, Refractory characterization, MOS-Sleep (Medical Outcomes Study Sleep Scale [13]), and Concomitant medications. Subjects who have a delay between their participation of the RESTFUL study and the CT-05 Extension Study (e.g. Arm 3 participants) will have some or all baseline assessment(s) repeated or re-affirmed.

16.8. Device training

Subjects will have already received training on device usage in The RESTFUL Study. For subjects in Arm 3, due to the delay between The RESTFUL Study and Arm 3, training reminders may be needed on proper usage of the device, including positioning of the devices on their legs. Instrument(s) designed for marking the human skin (body-marking pen or temporary tattoo) may be employed to mark the locations of electrode placement. Device calibration may be adjusted during device training based on subjective feedback from the subject.

16.9. Study Treatment Description

For Arms 1 and 3 (but not Arm 2), study subjects are instructed to self-administer treatment nightly as needed – typically after RLS symptoms start and before RLS symptoms become severe. The instructions for timing of device use may be adjusted based on the timing of RLS symptoms experienced by the subject. Once activated, a single session of treatment may run for approximately 30 minutes and then turn off automatically. Up to 120 total minutes per night may be used. If device battery life limits the number of uses per night, the following two timings of use may be prioritized: (1) at bedtime, to reduce symptoms and thus help with sleep initiation, and (2) when waking up in the middle of the night, to reduce symptoms and thus help with sleep re-initiation. Device intensity is typically set to the calibrated levels, but intensity may be increased if RLS symptoms are especially severe or decreased such as if RLS symptoms are mild.

16.10. Concomitant Medications and Treatments

Concomitant medications and treatments are tracked throughout the study based on subject reported data. For Arms 1 and 3 (but not Arm 2), study subjects are instructed to maintain a stable dose and schedule of all concomitant medications and treatments for RLS and of all concomitant medications and treatments that affect RLS symptoms, including antidepressants, sleep medications, or sedative antihistamines.

16.11. Leg movement data collection

The NTX100 Neuromodulation System may have the capability to collect data on leg movements, such as by using accelerometers and/or gyroscope sensors, while the subject is wearing the devices. These data may be collected during this study and used for subsequent analysis and development.

16.12. Evaluation and study assessment schedule

CONFIDENTIAL
All information contained in this document is confidential and is the sole property of Noctrix Health, Inc.
Any reproduction in part or whole without the written permission of Noctrix Health, Inc. is prohibited.

The following evaluation calls and/or visits are scheduled, as outlined in Tables 4 and 6 below. A window of +/- 4 days may be utilized on office follow-ups and a window of +/- 2 days for any phone follow-up, as needed. To reduce self-selection bias, is important to incentivize maximal participation of subjects from the RESTFUL Study in this study. One method to incentivize participation is to reduce the requirement for in-person visits, which can be especially challenging for subjects who do not live within a short distance of the clinical site.

Arms 1 and 3:

Evaluation 1: **In-person** visit for enrollment, including consent, screening, and assessments. For Arm 1, consent & screening will typically occur on the same visit as Eval 11 for the RESTFUL Study. If the subject's schedule does not permit completion of assessments during this office visit, then these assessments may be conducted via phone follow-up at any time within the 3-days following the office visit.

Evaluation 2: (2-wks after Eval. #1) Phone follow-up to assess RLS symptoms, NTX100 use and assess the occurrence of adverse events and/or changes to daily medications.

Evaluation 3: (4-wks after Eval #1) Phone follow-up to assess RLS symptoms, NTX100 use, and assess the occurrence of adverse events and/or changes to daily medications.

Evaluation 4: (8-wks after Eval #1) **In-person visit or Phone call*** follow-up to assess RLS symptoms, NTX100 use, and assess the occurrence of adverse events and/or changes to daily medications. Device replacement* and device log analysis may occur at this Evaluation, but this is not a strict requirement.

Evaluation 5: (12-wks after Eval #1) Phone follow-up to assess RLS symptoms, NTX100 use, and assess the occurrence of adverse events and/or changes to daily medications.

Evaluation 6: (16-wks after Eval #1) **In-person visit or Phone call*** follow-up to assess RLS symptoms, NTX100 use, and assess the occurrence of adverse events and/or changes to daily medications. Device replacement* and device log analysis may occur at this Evaluation, but this is not a strict requirement.

CONFIDENTIAL
All information contained in this document is confidential and is the sole property of Noctrix Health, Inc.
Any reproduction in part or whole without the written permission of Noctrix Health, Inc. is prohibited.

Evaluation 7: (20-wks after Eval #1) Phone follow-up to assess RLS symptoms, NTX100 use, and assess the occurrence of adverse events and/or changes to daily medications.

Evaluation 8: (24-wks after Eval #1) **In-person** follow-up to assess RLS symptoms, NTX100 use, and assess the occurrence of adverse events and/or changes to daily medications. Devices are returned at this time.

Evaluation 9: (32-wks after Eval #1) Phone follow-up to assess RLS symptoms, adverse events, and any changes to daily medications. Study exit occurs at this time.

** See Section 16.13 below for discussion of device replacement and in-person visits.*

In addition to these assessments, subjects will complete a Weekly Questionnaire each week throughout the 24-wk duration of device usage and a Bi-weekly Questionnaire every 2-weeks from Weeks 25-32.

The schedule of assessments are shown in Table 4 below. Additionally, the devices may automatically log quantitative compliance, usage data, and leg movement data throughout the duration of the study.

Table 4. Schedule of Assessments for Arms 1 and 3

All information contained in this document is confidential and is the sole property of Noctrix Health, Inc.
Any reproduction in part or whole without the written permission of Noctrix Health, Inc. is prohibited.

Schedule of assessments - Arms 1 and 3												
		Eval 1			Eval 2	Eval 3	Eval 4	Eval 5	Eval 6	Eval 7	Eval 8	Eval 9
		Day 0	Wks 1-24	Wks 25-32	Wk-2	Wk-4	Wk-8	Wk-12	Wk-16	Wk-20	Wk-24	Wk-32
		In-person	Remote	Remote	Remote	Remote	In-person or Remote**	Remote	In-person or Remote**	Remote	In-person	Remote
Assessment	Completed by											
Screening		X										
Consent		X										
Study entry questionnaire	Staff + subject	X										
Weekly questionnaire	Subject		X									
Biweekly questionnaire	Subject			X								
Review weekly questionnaire responses	Staff				X	X	X	X	X	X	X	
ConMeds	Staff	X			X	X	X	X	X	X	X	X
AE Monitoring/Reporting	Staff				X	X	X	X	X	X	X	X
IRLS	Staff + subject	X (Arm 3 only*)			X	X	X	X	X	X	X	X
PGI-I	Staff + subject				X	X	X	X	X	X	X	X
CGI-I	Investigator + subject						X		X		X	X
MOS-Sleep	Staff + subject	X (Arm 3 only*)				X	X	X	X	X	X	X
SF-36	Staff + subject	X									X	
Device completion questionnaire	Staff + subject										X	
Study exit questionnaire	Staff + subject											X
Device replacement	Staff + subject	X **					X **		X **			
		* For Arm 1, data from Eval 11 of the RESTFUL Study will be used, since both Evals typically occur on the same day										
		** Device replacement may not occur at these specific Evals; device log analysis & sponsor video call will be concurrent w/ device replacement										

Arm 2:

Evaluation 1: **In-person visit or Phone call** for enrollment, including consent, screening, and assessments. For Cohort 2A (see Section 16.2), consent & screening will typically occur in-person on the same visit as Eval 11 for the RESTFUL Study. If the subject's schedule does not permit completion of assessments during this office visit, then these assessments may be conducted via phone follow-up at any time within the 3-days following the office visit.

Evaluation 2: (8-wks after completion of the RESTFUL Study) Phone follow-up to assess RLS symptoms, any changes to daily medications, and adverse events.

Evaluation 3: (16-wks after completion of the RESTFUL Study) Phone follow-up to assess RLS symptoms, any changes to daily medications, and adverse events.

Evaluation 4: (24-wks after Eval #1) Phone follow-up to assess RLS symptoms, any changes to daily medications, and adverse events. Study exit.

CONFIDENTIAL
All information contained in this document is confidential and is the sole property of Noctrix Health, Inc.
Any reproduction in part or whole without the written permission of Noctrix Health, Inc. is prohibited.

As summarized in Table 5, enrollment to Arm 2 will either occur at either 0-wks (EVAL 1), 8-wks (EVAL 2), 16-wks (EVAL 3), or 24-wks (EVAL 4) after completion of the Restful Study, +/- 6-days. If enrollment occurs at 24-wks, the SF-36 and concomitant medication assessments will only be conducted once.

Table 5. Structure of Arm 2

Delay after RESTFUL study completion	Evals conducted
0-wks	1,2,3,4
8-wks	1,2,3,4
16-wks	1,3,4
24-wks	1,4

TABLE 2: SCHEDULE OF ASSESSMENTS FOR ARM 2

Schedule of assessments - Arm 2					
		Eval 1	Eval 2	Eval 3	Eval 4
		Day 0	Wk-8*	Wk-16*	Wk-24*
Assessment	Completed by	In-person or Remote	Remote	Remote	Remote
Screening		X			
Consent		X			
Study entry questionnaire	Staff + subject	X			
ConMeds	Staff	X	X	X	X
AE Monitoring/Reporting	Staff		X	X	X
IRLS	Staff + subject		X	X	X
PGI-I	Staff + subject		X	X	X
CGI-I	Investigator		X	X	X
MOS-Sleep	Staff + subject		X	X	X
SF-36	Staff + subject	X			X
Study exit questionnaire	Staff + subject				X

* Duration after Eval 11 of The RESTFUL Study, not CT-4 enrollment [not all subjects will enroll in time to complete Wk-8 or Wk-16 timepoints]

16.13. Device replacement in Arms 1 and 3

The investigational device may include components that are consumable or semi-consumable and may be replaced on a periodic or as-needed basis. Such device component replacement may occur via an in-person exchange or via delivery to the subject. At entry to Arm 1, subjects may continue using investigational device components from the RESTFUL Study prior to replacement – which may require one additional visit for in-person exchange or may be conducted via delivery to the subject.

Typically, at Evals 4 and 6, stimulation unit replacement will occur and device log data will be downloaded to assess quantitative compliance and usage data and follow-up with the subject regarding usage data. To facilitate this process, subjects will be encouraged to attend an in-person visit. For subjects who live further from the site and/or cannot easily travel to the site, replacement device components will be delivered to the subject and the subject will return used stimulation units to the site for device log data analysis. For these subjects, the Eval may be split into two parts, where the second part is conducted upon receipt of stimulation units at the site and involves a call to follow-up with the subject regarding usage data.

Each time new stimulation units are provided to a subject during this study, they must be programmed with the specific calibrated settings corresponding to that subject.

16.14. Administration of assessments

- **PGI-I** (Patient Global Impressions – Improvement Scale). The subject will determine the ratings, and the examiner should be available to clarify any misunderstandings the subject may have about the questions. The examiner or the subject may mark the subject's answers on the form.
- **CGI-I** (Clinical Global Impressions – Improvement Scale). The CGI-I is completed by the Investigator after conducting a short interview with the subject regarding their RLS symptoms over the past week compared to baseline. The Investigator will not be permitted access to the subject's IRLS responses but will be permitted access to the subject's PGI-I responses.
- **IRLS.** The subject will determine the ratings, and the examiner should be available to clarify any misunderstandings the subject may have about the questions. The examiner or the subject may mark the subject's answers on the form.
- **MOS-Sleep.** The subject will determine the ratings, and the examiner should be available to clarify any misunderstandings the subject may have about the questions. The examiner or the subject may mark the subject's answers on the form.

CONFIDENTIAL
All information contained in this document is confidential and is the sole property of Noctrix Health, Inc.
Any reproduction in part or whole without the written permission of Noctrix Health, Inc. is prohibited.

- **SF-36.** The subject will determine the ratings, and the examiner should be available to clarify any misunderstandings the subject may have about the questions. The examiner or the subject may mark the subject's answers on the form.
- The **Weekly Questionnaire** will be completed by the subject.
- The **Bi-weekly Questionnaire** will be completed by the subject every 2 weeks during Weeks 25-32 of Arms 1 and 3.
- **Subject characterization** will be completed by the examiner (site staff) based on information from the subject. Subject characterization information may be transferred from the database for the RESTFUL Study, if such information is available and has not changed.
- **Medical history** will be completed by the examiner (site staff) based on information from the subject. Medical history information may be transferred from the database for the RESTFUL Study, if such information is available and has not changed.

16.15. Study Exit

Study exit will occur at the conclusion of Eval #9. At study exit, the Investigator/coordinator will complete the study exit eCRF. Adverse events unresolved at study exit will be followed by the Investigator until resolution occurs or at least 30 days after the subject's participation in the study is complete.

16.16. Subject Discontinuation

A subject may be removed from the study prior to completion for any of the following reasons:

- Voluntary withdrawal of consent
- Adverse event preventing further study participation
- Investigator believes risk of further subject participation outweighs benefit
- Persistent non-compliance or lost to follow-up
- Pregnancy
- Subject no longer meets inclusion criteria
- [For arms 1 and 3 only] Subject makes a significant change to dosage of medications known to interfere with sleep or RLS symptoms, including sleep medications, antidepressants, sedative antihistamines.

CONFIDENTIAL
All information contained in this document is confidential and is the sole property of Noctrix Health, Inc.
Any reproduction in part or whole without the written permission of Noctrix Health, Inc. is prohibited.

- [For arms 1 and 3 only] Insufficient device availability (see 16.16.1 below)

16.16.1. Discontinuation related to device availability

As noted in Section 11, the investigational device may include components that are consumable or semi-consumable and may be replaced on a periodic or as-needed basis. In the unlikely event that investigational device supply becomes insufficient to replenish the consumable components of the devices for all subjects enrolled in the study, enrollment will be paused and device replacement will continue in a normal manner until the device supply for the study is exhausted (or nearly exhausted). At that point, remaining subjects will be removed from the study at their next scheduled device replacement timepoint.

Additionally, it is possible that some patterns of device use or misuse may lead to a reduction in the functional lifespan of the investigational device below 8-weeks. If this is suspected for a specific subject, that subject may be removed from the study.

The Investigator or research staff will complete a study exit form in the eCRF for any subject who prematurely discontinues from the study. If discontinuation was the result of an AE, the AE will also be recorded in the eCRF.

Upon discontinuation, subjects will receive partial compensation commensurate with their completed Phases.

16.6. Study Termination

The Sponsor may terminate the study as a whole or at individual study sites under the following circumstances:

- Suspicion of risk to subjects, including occurrence of high rate of known AEs or unexpectedly high rate of unexpected AEs
- Poor site compliance with the study protocol
- Inadequate site enrollment
- Obtaining new scientific knowledge that shows that the study is no longer valid or necessary
- Persistent non-compliance with IRB or regulatory requirements
- Persistent failure to comply with obligations arising from the clinical trial agreement

CONFIDENTIAL
All information contained in this document is confidential and is the sole property of Noctrix Health, Inc.
Any reproduction in part or whole without the written permission of Noctrix Health, Inc. is prohibited.

- Insufficient availability of investigational devices
- Other business reasons (e.g., insolvencies or business entity liquidation)

The sponsor will document reasons for study suspension and notify relevant site Investigators and governing IRBs. If suspension occurred because of a safety issue, all Investigators will be notified. When terminating the study, the sponsor and Investigator will assure that adequate consideration is given to the protection of the subjects' interests.

17. Study Endpoints and Analysis Overview

17.1. Treatment of data from the RESTFUL Study

CT-05 is an extension of the RESTFUL Study and Arms 1 and 2 involve a direct roll-over from the RESTFUL Study into CT-05. Therefore, data from the RESTFUL Study (CT-04) will be used to define the Baseline for Arms 1 and 2 and may be used to define subgroup analyses and demographic characterization.

17.2. Efficacy

17.2.1. Primary Efficacy Endpoint

Arm 1: The primary outcome measure will be responder rate on the CGI-I scale at Week 24 of Arm 1 relative to entry to the RESTFUL Study.

Arm 3: The primary outcome measure will be responder rate on the CGI-I scale at Week 24 of Arm 3 relative to CT-05 study entry.

Responder rate for the 7-point CGI-I scale will be defined as the proportion of responses of "Much Improved" or "Very Much Improved".

17.2.2. Key Secondary Efficacy Endpoints

Arm 1:

1. PGI-I responder rate (defined as for CGI-I) at Wk-24 relative to entry to the RESTFUL Study.
2. Mean reduction in IRLS score at Week 24 of Arm 1 relative to entry to the RESTFUL Study.
3. Mean reduction in MOS-II score at Week 24 of Arm 1 relative to entry to the RESTFUL Study.
4. Mean reduction in MOS-I score at Week 24 of Arm 1 relative to entry to the RESTFUL Study.
5. Frequency of RLS symptoms (based on IRLS question #7) at Week 24 of Arm 1 relative to entry to the RESTFUL Study.

Arm 3:

1. PGI-I responder rate (defined as for CGI-I) at Week 24 relative to CT-05 study entry.
2. Mean reduction in IRLS score at Week 24 of Arm 1 relative to CT-05 study entry.
3. Mean reduction in MOS-II score at Week 24 of Arm 1 relative to CT-05 study entry.
4. Mean reduction in MOS-I score at Week 24 of Arm 1 relative to CT-05 study entry.
5. Frequency of RLS symptoms (based on IRLS question #7) at Week 24 of Arm 1 relative to CT-05 study entry.

Definition and instructions for calculation of the Medical Outcomes Study Sleep Problems Index I and II are described in [13].

17.2.3. Efficacy Analysis

A Statistical Analysis Plan (SAP) will be developed prior to completion of the first subject in Arm 1 or 3, which will include a plan for efficacy analysis. The following analyses are planned:

- Primary statistical analysis may be conducted separately for subjects in Arms 1 and Arm 3. Additional pooled analysis across Arms 1 and 3 may also be conducted.
- Subgroup analysis form Arms 1 and 3 may be conducted based on the following criteria:
    - Responders at Week-8 of The RESTFUL Study, as defined by CGI-I of 1 or 2.
    - Non-responders at Week-8 of The RESTFUL Study, as defined by CGI-I of 3 or above.
- Missing data may be addressed with the use of multiple imputation.
- Appropriate statistical techniques will be employed to test statistical significance.
- If there is success on the primary endpoint, then secondary endpoints will be tested in order, according to the fixed-sequence method, until failure.

The analysis cohort for the primary efficacy endpoint are subjects who are enrolled and undergo the assigned treatment.

The Per Protocol analysis will exclude subjects for whom one or more of the following apply:

1. Dropout or removal from the study.
2. Incomplete or missing data for the endpoint.

CONFIDENTIAL
All information contained in this document is confidential and is the sole property of Noctrix Health, Inc.
Any reproduction in part or whole without the written permission of Noctrix Health, Inc. is prohibited.

3. Clinically significant change in dose or schedule of medication that affects RLS symptoms .
4. New medical information available after randomization indicates an exclusion criterion.
5. Other protocol deviation occurs that is deemed clinically significant by the investigator.
6. Device usage at or after bedtime on fewer than two-thirds of the nights with RLS symptoms at or after bedtime, as reported by the subject on the Weekly Questionnaire assessments.
7. Missing two or more follow-ups (Follow-ups completed out-of-window are not considered "missing")
8. Zero total device uses.

17.2.4. Additional efficacy analyses

Comparison of Arm 1 (investigational device) to Arm 2 (control)

Arm 1 and Arm 2 both cover the same period of time – the 24-wks after completion of the RESTFUL Study). Arm 1 involves continual open-label usage of the investigational device throughout this time period, whereas Arm 2 (control) involves no usage of the investigational device. Comparison of the data from these arms will be used to assess how continued use of the devices over 24-weeks impacts RLS symptoms, sleep, and medication use relative to the typical standard of care over 24-weeks.

- For Arm 2, baseline will be defined as entry to CT-04 Restful Study and similar analyses will be conducted as in Arm 1.
- Results from Wk-24 of Arm 2 (no device usage) will be compared to results from Wk-24 of Arm 1 (device usage), to assess the effect of continued device usage on the following outcomes:
  - Changes in RLS symptoms, as measured by CGI-I, PGI-I, and IRLS
  - Changes in Sleep, as measured by MOS-II and MOS-I
  - Changes in quality of life, as measured by SF-36
  - Changes in the dose and type of concomitant medications prescribed and/or administered to treat RLS

Changes to symptom severity over time

- Linear regression of PGI-I over all weeks of Active treatment*
- Linear regression of IRLS over all weeks of Active treatment*

CONFIDENTIAL
All information contained in this document is confidential and is the sole property of Noctrix Health, Inc.
Any reproduction in part or whole without the written permission of Noctrix Health, Inc. is prohibited.

- Linear regression of Frequency of RLS symptoms over all weeks of Active treatment* (based on IRLS question #7)
- Linear regression of MOS-II over all weeks of Active treatment*
- Linear regression of MOS-I over all weeks of Active treatment*

** For Arm 1, this starts at the beginning of Active treatment in the RESTFUL Study. For Arm 3, this starts at entry to Arm 3.*

Additional subgroup analyses

- Subjects assigned to Sham control for Weeks 1-4 of the RESTFUL Study
- Subjects assigned to Active treatment for Weeks 1-4 of the RESTFUL Study

17.3. Safety

17.3.1. Safety Endpoints

The safety endpoint will be a descriptive analysis of adverse events (AEs) for both study arms, classified and tabulated by seriousness, relationship to the device, and severity.

17.3.2. Safety Analysis

Frequency charts of adverse events occurring at any time during the study will be produced. The proportion of subjects in Arms 1 and 3 reporting one or more adverse events between study entry and Week 24 will be pooled and tabulated. Similar analyses will be done for SAEs and device-related SAEs. Safety analysis will include all subjects who were treated. A Statistical Analysis Plan (SAP) will be developed prior to completion of the first subject in Arm 1 or 3, which may include additional plans for safety analysis.

17.4. Compliance

Compliance will be assessed during the first 24-wks of Arm 1 and Arm 3. Subjects will be instructed to use devices each nights that they report experiencing RLS symptoms. Therefore, percentage compliance with device usage will be calculated using the following formula:

100%(nights of compliant use)/(total nights with patient-reported RLS symptoms)*

All information contained in this document is confidential and is the sole property of Noctrix Health, Inc.
Any reproduction in part or whole without the written permission of Noctrix Health, Inc. is prohibited.

- Nights of compliant use will be measured based on digital tracking of the timing and duration of each NPNS device usage. In cases where digital tracking logs are incomplete or unavailable, the data will not be included in the compliance analysis.
- A "*night of compliant use*" will be defined as a minimum of 25 minutes of active NPNS stimulation between noon and noon during an Assessment week.
- "*Total assessment nights with RLS symptoms*" will be defined as the total nights spent by all subjects in Assessment weeks when the subject reports RLS symptoms on the Weekly questionnaire.
- If a subject is discontinued from the study for reasons unrelated to subject response to treatment, compliance data will only be assessed up until the time of discontinuation. Such reasons could include lack of device availability, personal reasons, or adverse event unrelated to the study device.

17.5. Exploratory Analysis

Additional statistical analysis that is exploratory in nature may be performed with CT-05 data alone and/or with pooled data from CT-05 and from the RESTFUL Study.

18. Remote procedures

During and after the COVID-19 pandemic, replacing some in-person visits with remote interactions may be necessary, for example to reduce risk to subjects and/or to comply with local, state, or federal regulations. In such cases, the study records will indicate which in-person visits were replaced with remote interactions. The allowable study duration may be expanded as needed to allow delay intervals for mailing and receiving programmed devices.

In such cases, one of more of the following approaches may be taken:

1. Remote calibration and training. Remote calibration and training, where possible, will be coordinated by mailing a calibration/training device to the subject, conducting calibration and training via a video call using the calibration/training device, and then mailing programmed

CONFIDENTIAL
All information contained in this document is confidential and is the sole property of Noctrix Health, Inc.
Any reproduction in part or whole without the written permission of Noctrix Health, Inc. is prohibited.

devices to the subject using the settings determined during the calibration. Alternatively, the same devices used in the study may be used for calibration/training.

2. Remote follow-up visits. Remote follow-up visits, where possible, will be completed via call or video call, during which the required study assessments will be administered.
3. Devices may be returned using a pre-paid mailing label, as needed.

4. Remote consent. Remote consent procedures will follow the recommendations set forth in the "FDA Guidance on Conduct of Clinical Trials of Medical Products during COVID-19 Public Health Emergency" [14]. For example, or more of the following procedures listed in the guidance will be used to confirm consent:
   a. eConsent via a compliant digital platform;
   b. confirmation of consent by an impartial witness via three-way call or video conference;
   c. remote explanation of consent followed by in-person signature of informed consent document (to minimize duration of in-person interaction).

19. References

1. Allen, R.P. et al. Restless legs syndrome: diagnostic criteria, special considerations, and epidemiology. A report from the restless legs syndrome diagnosis and epidemiology workshop at the National Institutes of Health. Sleep Med. (2003). doi: 10.1016/s1389-9457(03)00010-8
2. 2012 Revised IRLSSG Diagnostic Criteria for RLS. Available at: http://irlssg.org/diagnostic-criteria
3. Allen, R. P. *et al.* Restless legs syndrome prevalence and impact: REST general population study. *Arch. Intern. Med.* (2005). doi:10.1001/archinte.165.11.1286
4. Silber, M.H. Treatment of restless legs syndrome and periodic limb movement disorder in adults. In: UpToDate, Post, TW (Ed), UpToDate, Waltham, MA, 2020
5. García-Borreguero, D. & Williams, A. M. Dopaminergic augmentation of restless legs syndrome. *Sleep Medicine Reviews* (2010). doi:10.1016/j.smrv.2009.11.006

CONFIDENTIAL
All information contained in this document is confidential and is the sole property of Noctrix Health, Inc.
Any reproduction in part or whole without the written permission of Noctrix Health, Inc. is prohibited.

6. Lipford, M.C. and Silber, M.H. Long-term use of pramipexole in the management of restless legs syndrome. Sleep Med. 2012; 13: 1280–1285

7. Hening, W.A. et al. An update on the dopaminergic treatment of restless legs syndrome and periodic limb movement disorder. Sleep (2004). doi: 10.1093/sleep/27.3.560

8. Cornelius, J.R, Tippmann-Peikert, M., Slocumb, N.L., Frerichs, C.F., Silber, M.H. Impulse control disorders with the use of dopaminergic agents in restless legs syndrome: a case-control study. Sleep. 2010 Jan;33(1):81-7.

9. FDA Drug Safety Communication 12-19-2019. Available at: https://www.fda.gov/drugs/drug-safety-and-availability/fda-warns-about-serious-breathing-problems-seizure-and-nerve-pain-medicines-gabapentin-neurontin.

10. Silber, M. H. *et al.* The Appropriate Use of Opioids in the Treatment of Refractory Restless Legs Syndrome. *Mayo Clin. Proc.* (2018). doi:10.1016/j.mayocp.2017.11.007

11. FDA Center for Devices and Radiological Health Guidance Jan 2006. Information sheet guidance for IRBs, clinical investigators, and sponsors: Significant Risk and Nonsignificant risk medical device studies. Available at: http://www.fda.gov/downloads/RegulatoryInformation/Guidances/UCM126418.pdf

12. Walters AS, LeBrocq C, Dhar A, et al. Validation of the International Restless Legs Syndrome Study Group rating scale for restless legs syndrome. *Sleep Med*. 2003;4(2):121-132. doi:10.1016/s1389-9457(02)00258-7

13. Hays, R. D., & Stewart, A. L. (1992). Sleep measures. In A. L. Stewart & J. E. Ware (eds.), Measuring functioning and well-being: The Medical Outcomes Study approach (pp. 235-259), Durham, NC: Duke University Press.

14. FDA Guidance on Conduct of Clinical Trials of Medical Products during COVID-19 Public Health Emergency. Available at: https://www.fda.gov/media/136238/download.

CONFIDENTIAL
All information contained in this document is confidential and is the sole property of Noctrix Health, Inc.
Any reproduction in part or whole without the written permission of Noctrix Health, Inc. is prohibited.
